# A Binary Logistic Regression Model as a Tool to Predict Craft Beer Susceptibility to Microbial Spoilage

**DOI:** 10.3390/foods10081926

**Published:** 2021-08-19

**Authors:** Magaly Rodríguez-Saavedra, Karla Pérez-Revelo, Antonio Valero, M. Victoria Moreno-Arribas, Dolores González de Llano

**Affiliations:** 1 Department of Food Biotechnology and Microbiology, Institute of Food Science Research, CIAL (CSIC-UAM), C/Nicolás Cabrera 9, 28049 Madrid, Spain; 40204047@pronabec.edu.pe (M.R.-S.); karlaperez9310@gmail.com (K.P.-R.); victoria.moreno@csic.es (M.V.M.-A.); 2 Department of Food Science and Technology, Campus de Rabanales, University of Cordoba, Edificio Darwin, 14014 Córdoba, Spain; bt2vadia@uco.es

**Keywords:** spoilage microorganisms, susceptibility prediction, antimicrobial hurdles, beer intrinsic factors, growth/no growth, model development

## Abstract

Beer spoilage caused by microorganisms, which is a major concern for brewers, produces undesirable aromas and flavors in the final product and substantial financial losses. To address this problem, brewers need easy-to-apply tools that inform them of beer susceptibility to the microbial spoilage. In this study, a growth/no growth (G/NG) binary logistic regression model to predict this susceptibility was developed. Values of beer physicochemical parameters such as pH, alcohol content (% ABV), bitterness units (IBU), and yeast-fermentable extract (% YFE) obtained from the analysis of twenty commercially available craft beers were used to prepare 22 adjusted beers at different levels of each parameter studied. These preparations were assigned as a first group of samples, while 17 commercially available beers samples as a second group. The results of G/NG from both groups, after artificially inoculating with one wild yeast and different lactic acid bacteria (LAB) previously adapted to grow in a beer-type beverage, were used to design the model. The developed G/NG model correctly classified 276 of 331 analyzed cases and its predictive ability was 100% in external validation. This G/NG model has good sensitivity and goodness of fit (87% and 83.4%, respectively) and provides the potential to predict craft beer susceptibility to microbial spoilage.

## 1. Introduction

Craft beer is a beverage made from water, yeast, malt, and hops, in most cases without filtration or pasteurization, with an original flavor and final notes making each craft beer unique. Currently, the most popular beer styles are India Pale Ale (IPA), Brown Ale, Pale Ale, Pale Lager, Pilsner, Amber Ale, Amber Lager, Dark Lager, Porter, Stout, Bock, Strong Ale, and Wheat beer according to the Guidelines of the Beer Judge Certification Program [1].

Beer has generally been considered as a microbiologically safe beverage due to its low pH, oxygen concentration and available nutrients, plus the presence of alcohol (up to 14% ABV) and hop-derived compounds [2,3]. Ethanol and hops interfere with essential cell membrane functions of microorganisms, the low pH hinders enzyme activity, the lack of nutrients and oxygen starves many potential pathogens, while elevated dissolved carbon dioxide lowers the pH, inhibits enzymes, affects cell membranes, and creates an anaerobic environment [4]. Despite the intrinsic antimicrobial hurdles of beer, certain microorganisms can proliferate in this environment and cause beer spoilage, reducing the shelf-life of the beer. Haze production, sedimentation, acidification, turbidity, ropiness, and off-flavors [5], or biogenic amines generation [6] are the most common defects. 

In brewing, most spoilage incidents are caused by Gram-positive bacteria since mainly lactic acid bacteria (LAB) have developed resistance mechanisms to the hop compound, and approximately half of the documented microbiological incidents have been attributed to secondary LAB contamination [7,8]. In craft brewing, the species with the highest spoilage incidence are *Lactobacillus brevis* and *Pediococcus damnosus;* although other detrimental species have also been reported such as *L. lindneri, L. paracasei, L. plantarum,* and some *Leuconostoc* sp. [9,10,11,12]. Wild yeasts can cause the generation of phenolic off-flavors, undesirable high alcohol content, turbidity, carbonation changes, as well as a decrease in beer body and final quality [7]. Craft beer spoilage incidents are an insidious and unsolved problem causing substantial economic loss to the industry [13]. 

The application of logistic regression models in food microbiology has been proposed over recent decades, as they enable modeling the boundary between the growth/no-growth (G/NG) of microorganisms when certain controlling factors are studied, particularly in food products on the edge of microbial stability [14,15]. In view of this, mathematical models to predict the probability of spoilage by microorganisms of cold-filled ready-to-drink beverages, either by *Acinetobacter calcoaceticus* or by *Gluconobacter oxydans* in response to various preservation systems or by *Saccharomyces cerevisiae*, *Zygosaccharomyces bailii,* and *Candida lipolytica* as a function of a beverage formulation were created by Battey et al. [16,17]. Likewise, the applicability of logistic regression approaches for reducing *Escherichia coli* O157:H7 populations, as a valuable tool for designing safe apple cider processes was validated by Uljas et al. [18]. Recently, Munford et al. [19], modelled the inactivation effect on *Lactobacillus brevis* DSM 6235 while retaining the brewer’s yeasts viability during their acid washing, and suggested that the validated predictive models may be used to define washing protocols reducing breweries waste and costs, as well as in the industrial environment.

G/NG models of specific spoilage microorganisms, which describe the influence of different environmental conditions on growth probability, were also performed to define product reformulations, maintaining shelf-stable products such as sauces [20]. G/NG models allow calculating the probability of a binary outcome G/NG as a linear function by a combination of predictor variables such as pH, ethanol, water activity, and time. They also provide a useful tool for the development of new sweets with lower content of preservatives, fat, and sugars [21].

Currently, craft breweries produce various beer styles within the same factory, but they usually do not know which ones are the most susceptible to microbial contamination. Despite multiple studies focusing on investigating the beer spoilage ability of microorganisms [9,11,22] there is increasing interest in models for predicting microbial beer susceptibility, especially in craft breweries. G/NG binary logistic regression models have the appropriate capacity and ability to incorporate any environmental factor and its interactions [23,24]. To the best of the authors’ knowledge, this is the first study to develop an accurate model to predict the susceptibility of craft beer to microbial spoilage. In the present study, G/NG binary logistic regression models to predict the probability associated with each value of the binary response and a stepwise procedure to select the most important beer component predictors were used. The G/NG binary logistic regression model to predict craft beer susceptibility to microbial spoilage was performed as a function of main beer physicochemical parameters, without considering the beer spoilage microorganism strains as variable to develop a tool for craft brewers to adjust certain physicochemical parameter to enhance microbial stability. 

## 2. Materials and Methods

### 2.1. Microorganisms and Adaptation Process to Beer-Type Beverage

Wild LAB strains: *Lactobacillus brevis* CIALBL1 (L1), *L. brevis* CIALBD1 (D1), *L. plantarum* CIALBF1 (F1), *L. paracasei* CIALB6 (B6), *Leuconostoc pseudomesenteroides* CIALB2 (B2), *L. citreum* CIALB1 (B1), *Pediococcus damnosus* CIALBF2 (F2), the collection strain *L. brevis* CECT216 (216), and the wild yeast strain *Dekkera bruxellensis* CIALH2 (H2) from the research group at the Food Science Research Institute (CIAL–CSIC, Madrid, Spain) collection were selected, based on their resistance or susceptibility to hop bitter substances in order to include different potential risks of beer spoilage (Table 1). These strains were identified by 16S rRNA amplification and their beer-spoilage abilities were previously assayed [22]. LAB were grown in de Man, Rogose, Sharpe (MRS) broth (Difco™, Bordeaux, France) under anaerobic conditions at 28 °C for 48 h, while H2 yeast strain was cultivated in yeast extract-peptone-dextrose (YPD) broth (Difco™, France) at 30 °C for 72 h. All strains were adapted to grow sequentially in three different beverages according to Rodriguez-Saavedra et al. [22]: beverage 1 (5.4% YFE, 5.8 IBU, 1.75% ABV, pH 5.22), beverage 2 (3.4% YFE, 7.8 IBU, 3.0% ABV, pH 4.85), and beverage 3 (1.7% YFE, 9.4 IBU, 4.0% ABV, pH 4.55), where YFE, yeast fermentable extract; IBU, international bitterness units and ABV, alcohol by volume. The three different beverages were inoculated serially with isolates at 5 × 10^5^ cell mL^−1^. First, inoculum was added to tubes containing beverage 1 and incubated at 28 °C until visible growth was attained (18–36 h). Then, the isolates were transferred to beverage 2, when visible growth occurred (24–48 h) they were inoculated into beverage 3 until visible growth was observed (48 h–7 days).

### 2.2. Determination of the Early Stationary Growth Phase

Inocula of each strain were adjusted to an initial cell density at DO_590nm_ = 1 (~10^8^ CFU mL^−1^ for bacteria and ~10^7^ CFU mL^−1^ for yeast) in 0.9% NaCl solution. Then, 100 µL were inoculated into 10 mL of beverage 3 in duplicate and incubated at 28 °C in anaerobic conditions. The culture growth was monitored by DO_590nm_ measurements at 9 h intervals using a spectrophotometer Specord^®^210 by WinASPECT^®^ PLUS software (V. 4.2, Analytik Jena AG, Jena, Germany) until three constant readings were obtained. 

### 2.3. Data Generation for the First Group of Samples

**Figure 1:**
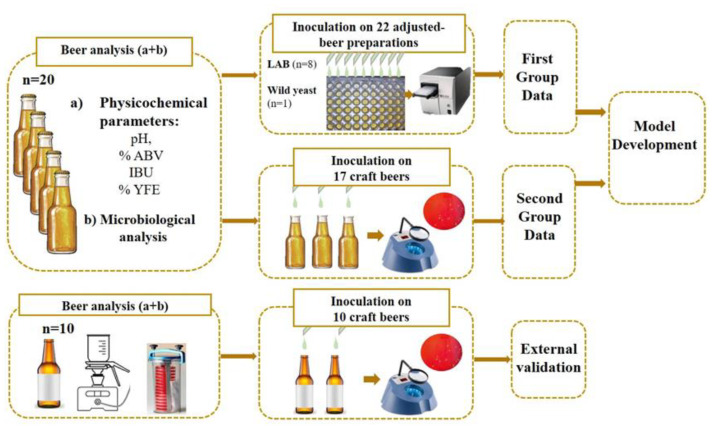
Workflow study scheme.

A workflow study scheme displays the main steps performed to build and validate the model is reached as shown in Figure 1.

#### 2.3.1. Microbial and Physicochemical Analysis of Craft Beers

Two different brands, named A and B, for each beer style: IPA, Brown ale, Pale ale, Amber ale, Porter, Stout, Bock, Strong ale, and Wheat beer, or for the non-alcoholic beers (<0.5% ABV) (*n* = 20) (Table 2) were purchased locally and stored at 4 °C. The membrane filtration technique described in Microbiological Control 2C [25], filtering 100 mL of beer per analysis, was used for LAB, acetic acid bacteria, and wild yeast detection in the commercial beer samples, using MRS medium; Lee’s multi-differential agar; and Lysine and MYGP with cupper medium, respectively, which were prepared and incubated according to Microbiological Control 5 [25]. Beer ethanol content (% ABV), pH, IBU, and % YFE were determined in triplicate according to Beer 4F, Beer 9, Beer 16, and Beer 23A protocols [25], respectively. All recorded data were processed using analysis of variance (ANOVA) with Statgraphics Centurion XVI software (V. 18, Statgraphics Technologies, Inc., The Plains, VA, USA) and the statistical significance was assessed by the Least Significant Difference (LSD) test, (*p* < 0.05). The information obtained from physicochemical parameters was used to define the levels of % ABV, pH, IBU, and % YFE in the adjusted-beer preparations. 

#### 2.3.2. Adjusted-Beer Preparation

Based on the pH, % ABV, % YFE, and IBU values of the beers determined in Section 2.3.1., study levels were created and 22 adjusted beers were prepared in order to evaluate the influence of each physicochemical parameter on microorganism growth. The 22 adjusted beers were prepared using selected beers bought on the market and modifying only one parameter while the other parameters remained unchanged. Firstly, to evaluate pH influence, a craft beer (11 IBU, 0.5% ABV, pH 4.25, and 1.45% YFE) was adjusted to six pH levels (3.2, 3.5, 3.8, 4.1, 4.4, and 4.7) using 7 M HCl or 7 M NaOH. Regarding ethanol influence, a non-alcoholic beer (11 IBU, 0.04% ABV, pH 4.18, and 2.4% YFE) was adjusted to six levels of % ABV (0.04%, 2.5%, 5.0%, 7.5%, 10.0%, and 12.0%) by addition of absolute ethanol. Bitterness units were evaluated by mixing a craft beer (10 IBU, 0.1% ABV, pH 4.2, and 2% YFE) with a non-alcoholic beer (52 IBU, 0.1% ABV, pH 4.6, and 3% YFE) to obtain six IBU levels (10, 15, 20, 30, 40, and 50). The preparation of these mixtures only caused minimal changes in the other parameters [22]. Finally, a non-alcoholic beer (9 IBU, 0.1% ABV, pH 4.20, and 0.76% YFE) was adjusted to four levels of % YFE (0.76%, 1.16%, 1.96%, and 2.36%) by addition of sterile maltose syrup. Before preparation, all the beers were sterilized by double filtration (0.45 µm). 

#### 2.3.3. G/NG Evaluation for the First Group of Samples

The effects of the six levels of pH, IBU, and % ABV, and the four levels of % YFE on the growth of the strains were investigated. For this, each of the strains (Table 1) was inoculated separately into the 22 adjusted beers in triplicate. Previously, the beer-adapted microorganisms (L1, D1, 216, F1, B6, B2, B1, F2, and H2) were harvested by centrifugation (3000 rcf, 5 min) from 50 mL of beverage, washed twice, and the pellets were suspended in 0.9% NaCl solution to prepare the inocula. The inoculation processes were made inside an anaerobic cabinet adding in triplicate 10 μL of inoculum into each well of 96 wells microplates containing 240 μL of each adjusted-beer prepared in order to reach 5 log CFU mL^−1^ [26]. Afterwards, microplates were sealed to minimize loss of volume and oxygen intake. For testing alcohol impact, 50 μL of inoculum was added into 1.2 mL of adjusted-beer in 2 mL Eppendorf tubes. Un-inoculated wells/tubes for each adjusted-beer were used as a blank.

The OD_590nm_ were measured in a BioTek SYnergy^TM^ HTX Multi-mode microplate reader, using the Gen5^TM^ 2.0 data analysis software (BioTek Instruments, Winooski, VT, USA) at the time and after 15 days of static incubation at 28 °C under anaerobic conditions. When growth was confirmed (if the difference between the OD_sample_ and OD_blank_ was consistently above three times the standard deviation of the signal of the blank) [20], value “1” was recorded, and value “0” if it was not. In this way, the dichotomous response variables were assigned. At the end of incubation, samples with value “1” were checked for their purity according to Rodriguez-Saavedra et al. [22]. Finally, detected G/NG values clearly anomalous, outliers, were excluded because they were considered to be cases in which environmental conditions were less severe prompting a decrease in the probability of growth, or vice versa [27]. 

### 2.4. Data Generation for the Second Group of Samples

#### 2.4.1. Selection of Commercial Craft Beers

Seventeen craft beers with no spoilage bacteria were selected (Figure 1) after the microbiological analysis (Section 2.3.1) of the commercial craft beers.

#### 2.4.2. G/NG Evaluation for the Second Group of Samples

Craft beers bottles were individually homogenized before opening and inoculated (5 × 10^3^ CFU mL^−1^) with each beer-adapted strain (L1, D1, 216, F1, B6, B1, F2, and H2) (Table 1) inside an anaerobic cabinet and in duplicate. Bottles were again closed inside the cabinet using a bottle capper tool and sterile crowns. These samples were incubated at 28 °C for 30 days and evidence of growth (turbidity, haze, ropiness, gas formation, or agglomeration) were visually assessed. Viable LAB and yeast cell counting were performed using the spot-plate technique at 0 and 30 days, on MRS agar supplemented with 10 ppm cycloheximide, 2 g L^−1^ maltose, and 0.04% chlorophenol red for LAB incubating under anaerobic conditions at 28 °C for 10 days, and YPD agar supplemented with 10 ppm cycloheximide and 0.022 g L^−1^ bromocresol green for wild yeast at 30 °C for 7 days. Value “1” was assigned in spoiled samples and in cases of microbial growth (if a difference of more than 1 log CFU mL^−1^ with the initial inoculum was detected) [28], and value “0” if it was not. Colonies were also checked by color and their morphological characteristic, microscopy, KOH and peroxidase tests. Out of the observed G/NG responses, outliers were detected and excluded.

### 2.5. Model Development

A model was built by merging the data from both groups of samples, in order to work with the greatest amount of data to enhance the model robustness and accuracy. The categories for the statistical analysis remained as described above.

A binary logistic regression model was developed to assess the probability of growth according to the equation: Logit (*P*) = ln (*P*/(1 − *P*)) = *f*, where *P* is the growth probability to be modeled, and *f* is defined by the following function *f* = *b*_0_ + *b*_1_ *F*_1_ + *b*_2_ *F*_2_ + … + *b*_n_ *F*_n_ + *b*_12_ *F*_1_ *F*_2_ + … + *b*_n−1,n_ *F*_n−1_ *F*_n_; in which *b* represents parameters to be fitted, and *F*_n_ represents the four factors (pH, IBU, % ABV, % YFE) used in the model.

Data were modeled to link a binary response variable G/NG of all assayed strains in the model to the set of the four physicochemical parameters through a polynomial expression incorporating the interactions among factors. Pearson’s correlation coefficient, tolerance, variance inflation factor, and the condition index, the most important statistical indexes for multi-collinearity diagnosis, were determined. Multi-collinearity analysis and binary logistic regression analysis were fitted in IBM^®^ SPSS^®^ Statistics 25.0 software (IBM SPSS, Inc., Chicago, IL, USA), and the confidence interval and level of significance were set at 95% and *p* < 0.05, respectively. The forward stepwise (Wald) method was selected to enter the factors, one by one, into the model. Predictive performance indexes and goodness-of-fit statistics were calculated: (i) omnibus test of model coefficient, (ii) the determination coefficient Nagelkerke R^2^, (iii) Hosmer–Lemeshow (HL) statistic; and (iv) SPSS classification table with a 95% confidence level. To visualize model predictions, graphical representations were built as contour plots considering the predicted cut-off probabilities of 0.9; 0.5; and 0.1. Furthermore, estimated growth probabilities were calculated for different levels of pH and % ABV. The established cut-off point for the model was 0.5, being *P* = probability of microbial growth.

### 2.6. Model Validation

External validation of the model was performed within the interpolation area [29] with additional data which was generated from the ninety G/NG evaluations from the other ten commercial craft beers. These beers were selected as they belonged to different beer styles and inoculated with each of the beer-adapted strains (L1, D1, 216, F1, B6, B2, B1, F2, and H2) following the method detailed in Section 2.4.2. Their physicochemical parameters were determined according to Section 2.3.1. The observed probabilities were determined for each beer tested using the G/NG results. After that, predicted probabilities were calculated using the model and compared with the observed probability to carry out a validation. According to the probability value of each tested beer and the cut-off points of the model, beers were classified as an “easy to spoil” beer if the probability was greater than the cut-off point (0.5); or as a “not easy to spoil” beer when the predicted probability was lower. Finally, the acceptability of the model was established taking into account if specificity (true negative ratio) and sensitivity (true positive ratio) had percentages close to 100%.

## 3. Results and Discussion

### 3.1. Adaptation of Microorganisms to Beer-Type Beverages and Determination of the Early Stationary Phase

The nine selected strains (Table 1) were able to adapt sequentially to the three beverages prepared with a gradual increasing of % ABV and IBU while reducing pH and % YFE. The beer-adapted cell sizes of *L. brevis* strains were considerably reduced (3–6 µm) in this beverage compared to the non-adapted strains, which grew on MRS media (10–12 µm). This fact is due to LAB having different resistance mechanisms to the intrinsic antimicrobial hurdles of beer, such as contact surface reduction. This finding is in concordance with the results reported by Zhao et al. [30] and Asano et al. [31]. 

Cultures in the early stationary phase were used since cultures in exponential growth phase are more likely to be susceptible to adverse conditions [32]. LAB reached the early stationary phase between 24–56 h in MRS media, while for *D. bruxellensis* this took 60 h in YPD media. However, the early stationary phase in beverage 3 for *D. bruxellensis*, *L. brevis*, *L. plantarum*, *P. damnosus*, and *L. paracasei* was reached at 79–85 h, while for *L.* *pseudomesenteroides*, and *L. citreum* this took 91 h. These periods are reasonable given that beer-type beverages generate stress conditions for microbial cells that can lead to an extension of their stages of adaptation [30]. 

### 3.2. Model Data

#### 3.2.1. First Group Data

The results of the physicochemical analysis of the craft beers (Section 2.3.1) are shown in Table 2. Alcohol content in the beer samples ranged from 0.3% to 12% ABV and the minimum value corresponded to the non-alcoholic beer A, while Bock B and Stout B beers showed the maximum value. This % ABV range mostly covered the alcohol scale described by Strong and England [1] that had a maximum theoretical value of 14% ABV. The pH values ranged from 3.14 (Weissbier-Wheat B beer) to 4.70 (IPA B beer), and non-significant differences (*p* < 0.05) were observed among samples with pH from 4.06 to 4.37 despite belonging at different beer styles. The % YFE ranged from 0.18% to 3.05%, and significant differences (*p* < 0.05) among samples belonging at the same beer style were found. In the same way, IBU extended from 10 IBU (Bock B beer) to 75 IBU (IPA B beer) and significant differences (*p* < 0.05) were observed in samples belonging to the same beer style. All these results were used to establish study levels for each physicochemical parameter (pH, % ABV, IBU, and % YFE) and to prepare the 22 adjusted beers in order to evaluate the influence of each parameter on microorganism growth.

The results of G/NG evaluation per physicochemical parameter indicated that all strains were able to grow in the adjusted-beers with % ABV from 0 to 5, pH from 4.1 to 4.7; bitterness from 10 to 30 IBU, and in all tested % YFE levels (from 0.76 to 2.36) (Supplementary Table S1) (No. assay 1–197). Eight strains were able to grow at pH 3.8, six strains at pH 3.5, and six strains under extreme levels of bitterness (40–50 IBU), while in high alcohol beers, four strains grew at 7.5–10% ABV. *Dekkera* strain was the only one able to grow at extreme alcohol content and pH conditions (12% ABV and pH 3.2). One outlier was only detected and excluded from first group G/NG data to model development.

#### 3.2.2. Second Group Data

**Figure 2:**
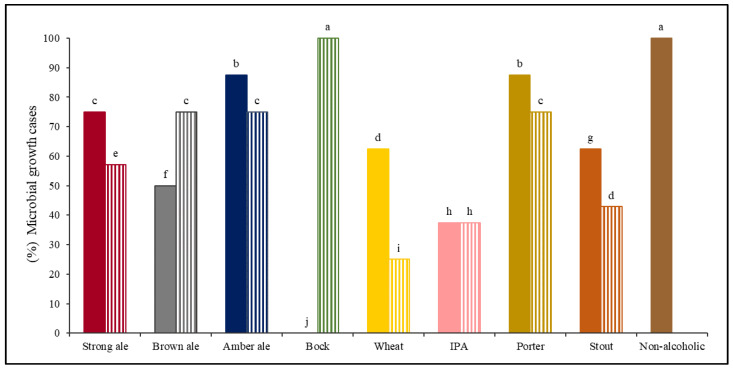
Percentage of microbial growth cases in the seventeen craft beers: Strong ale (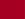
A; 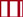
B), Brown ale (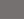
A; 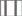
B), Amber ale (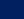
A; 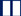
B), Bock (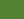
A; 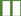
B), Wheat beer (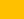
A; 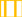
B), IPA (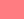
A; 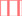
B), Porter (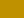
A; 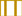
B), Stout (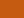
A; 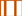
B), and Non-alcoholic beer (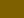
A). Percentage of microbial growth cases with different lowercase letters (a–j) above each column are significantly different (*p* < 0.05), using the LSD test. A and B identify two different brands for each beer style.

Data from 17 out of the 20 commercial craft beers analyzed were used. A microbial growth case (MGC) was assigned to spoiled beer samples according to G/NG evaluation (Section 2.4.2). The % MGC observed in these beers are shown in Figure 2. The most susceptible beers were the non-alcoholic A and Bock B beers, which allowed the growth of all inoculated strains (100% MGC), while no microorganism was able to grow in Bock A beer (0% MGC) probably due to its higher alcohol content (12% ABV) (Table 2). On the other hand, for the hoppiest beers which contain the highest concentration of antimicrobial substances (i.e., iso-α-acids), therefore higher values of IBU, only hop-tolerant strains were able to grow, as in the case of both the IPA beers (~75 IBU). Dekkera strain was able to grow in all beers (except in Bock A beer), exhibiting a strong growth, which could be due to the relatively high content of fermentable sugars available for this strain.

Non-alcoholic A beer was expected to have 100% MGC due to its physico-chemical parameters: very low alcohol content (0.3% ABV), a pH value that allows the growth of several microorganisms (pH = 4.22), low concentration of iso-α-acids (11 IBU), and the highest value of fermentable extract (2.37% YFE). In the case of Wheat beers, a lower % MGC was observed for Wheat B beer despite it contains lower alcohol content and concentration of iso-α-acids, and a higher value of fermentable extract than Wheat A beer, showing the inhibitory effect of the low pH (pH = 3.14) on microbial growth, clearly. A similar effect of the low pH on microbial growth was observed in Brown Ale beers, where Brown Ale A beer showed lower % MGC than Brown Ale B beer, despite the first contained less alcohol and IBU.

Significant differences in microbial cases were observed between beers of the same style, except for IPA style. This fact is due to a beer style has a wide range of values in each physical-chemical parameter. Moreover, many breweries change these values according to the desired flavor. Results of the G/NG evaluation on these commercial beers are available in Supplementary Table S1 (No. assay 198–331). Out of these observed G/NG responses from second group, only two outliers were detected and excluded.

### 3.3. Model Development

The model developed was based on combination of G/NG data from the two beer groups (Section 2.3.3 and Section 2.4.2) whose results are available in the Supplementary Table S1. A polynomial logistic regression model was performed to describe the influence of a binary response variable G/NG on the main beer physicochemical parameters, (pH, BU, % ABV, and % YFE) which is expressed by the following function:

$$ln\left(\frac{P}{1-P}\right)=-9.608-0.346\%\;\mathrm{ABV}-0.042\;\mathrm{IBU}+3.161\;\mathrm{pH}$$

In the equation, *P* is the growth probability (*P* takes values between 0 and 1) and the factors of pH, % ABV, and IBU were found to have an impact in predicting microbial growth, while % YFE was not significant for beer susceptibility to microbial spoilage. The binary logistic regression model, having just three parameters, can properly describe the G/NG boundary of spoilage microorganisms in craft beers, thus being easy to use and implement by brewers to accurately predict adequate beers’ formulations.

#### 3.3.1. Multi-Collinearity Analysis

In this study, the tolerance values were greater than 0.4 and <1, and the variance inflation factor values lower than 2.3, indicating no multi-collinearity was shown among the variables of the model. As a value of tolerance <0.10 and/or a variance inflation factor value >2.5 are indicative of multi-collinearity concern [33], our findings allowed continuing with the modeling process.

#### 3.3.2. Evaluation of Model Performance

The predictive capacity of the fitted model was analyzed by using Statgraphics Centurion X with a cut-off point at 0.5 (*P* = probability of microbial growth). A summary explaining the evaluation of the performance of the model is given in Table 3. The model goodness-of-fit was assessed with the Nagelkerke R^2^ value [34] and the HL goodness-of-fit test [35] and the *p*-value was >0.05, proving model showed an adequate fit level.

The Classification Table to evaluate the distribution of observed and predicted values by the model reached a prediction accuracy rate of 83.4% indicating an adequate prediction (Table 3). In addition, the model values of sensitivity and specificity were a 87% and 71%, respectively (Table 3) indicating a good acceptability of the model [36].

Table 3 also shows the variables that contribute to the model, their regression coefficients, Wald values, associated statistical significances, and odds ratio (OR). The OR values showed that pH had the greatest influence on the model response variable, with the OR values for pH > 1. This fact indicates that an increase in this variable value could increase the risk of microbial growth in beer. The model OR values for bitterness and alcohol content were <1, hence an increase in these variable values reduces the risk of beer spoilage.

This lower influence of % ABV and IBU than pH values on the microbial growth probability established by the model is related to high tolerance to alcohol content and IBU of some *Lactobacillus* spp. but they were not able to grow at low pH, despite being able to regulate their intracellular pH against acidic conditions. Moreover, a synergistic effect of low pH with the antibacterial activity of hop-derived bitter compounds has been demonstrated, as a small decrease of pH in beer (from 4.4 to 4.2) induces an increase of over 50% in the antibacterial activity of hop-derived bitter compounds [37,38]. Menz et al. [39] also found that pathogens growth in alcohol-free beers was prevented by lowering the pH from 4.3 to 4.0, as well as in higher ethanol beers possessing higher stability against beer microbial spoilage. 

**Figure 3:**
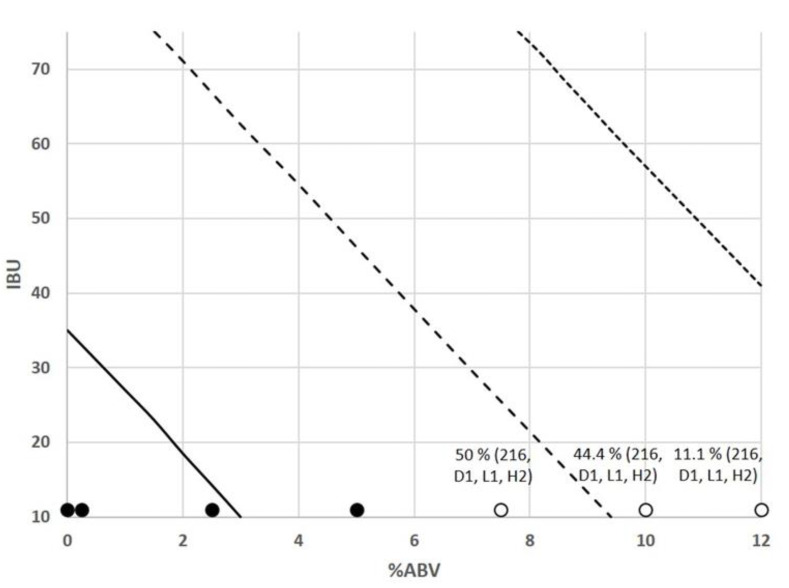
Contour plots for the observed growth responses and predicted probabilities (P = 0.1 (⋯⋯), P = 0.5 (- - -), and P = 0.9 (—)) for the microbial spoilage of craft beer as a function of % ABV and IBU. Values of pH and % YFE were set at 4.2 and 2.37, respectively. (●) P = 1 (growth), (○) P = 0 (no growth). Percentages indicate the proportion of strains showing growth, being described between brackets.

**Figure 4:**
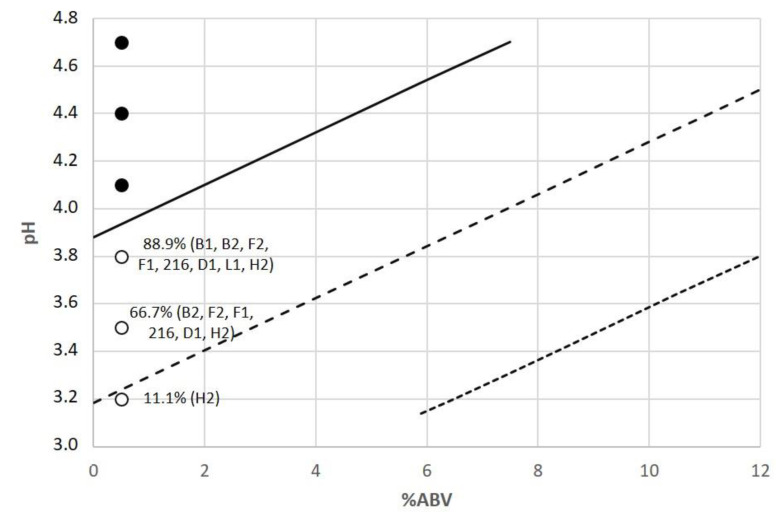
Contour plots for the observed growth responses and predicted probabilities (P = 0.1 (⋯⋯), P = 0.5 (- - -), and P = 0.9 (—)) for the microbial spoilage of craft beer as a function of % ABV and pH. Values of IBU and % YFE were set at 11 and 1.45, respectively. (●) P = 1 (growth), (○) P = 0 (no growth). Percentages indicate the proportion of strains showing growth, being described between brackets.

**Figure 5:**
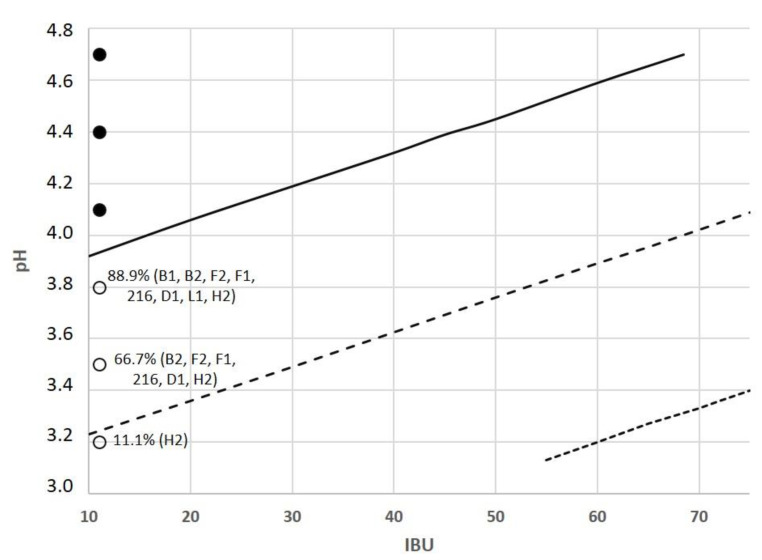
Contour plots for the observed growth responses and predicted probabilities (P = 0.1 (⋯⋯), P = 0.5 (- - -), and P = 0.9 (—)) for the microbial spoilage of craft beer as a function of IBU and pH. Values of % ABV and % YFE were set at 0.5 and 1.45, respectively. (●) P = 1 (growth), (○) P = 0 (no growth). Percentages indicate the proportion of strains showing growth, being described between brackets.

Logistic regression model predictions were performed to evaluate the model performance and to assess the growth predicted probabilities at 0.9; 0.5; and 0.1 levels for each pair of the main beer hurdle factors (Figure 3, Figure 4 and Figure 5). The evolution of the probability of microbial growth as a function of % ABV and IBU (Figure 3) showed that it was higher at the lowest IBU and % ABV levels, and the growth percentage decreases as the % ABV increases. The probability of microbial growth as a function of pH and % ABV (Figure 4) shows that at the lowest % ABV level, as the pH decreases the growth percentage decreases, being these probabilities lower in sour beers with pH values around 3.5 than in most beer styles (pH from 4.1 to 4.5). 

Likewise, the effect of pH as a function of IBU on the microbial growth (Figure 5) shows that at the lowest IBU as the pH decreases the microbial growth percentage decreases. These results of cross-sections of the G/NG interfaces show the model presented a good fit to the data observed since growth observations have been correctly classified.

**Figure 6:**
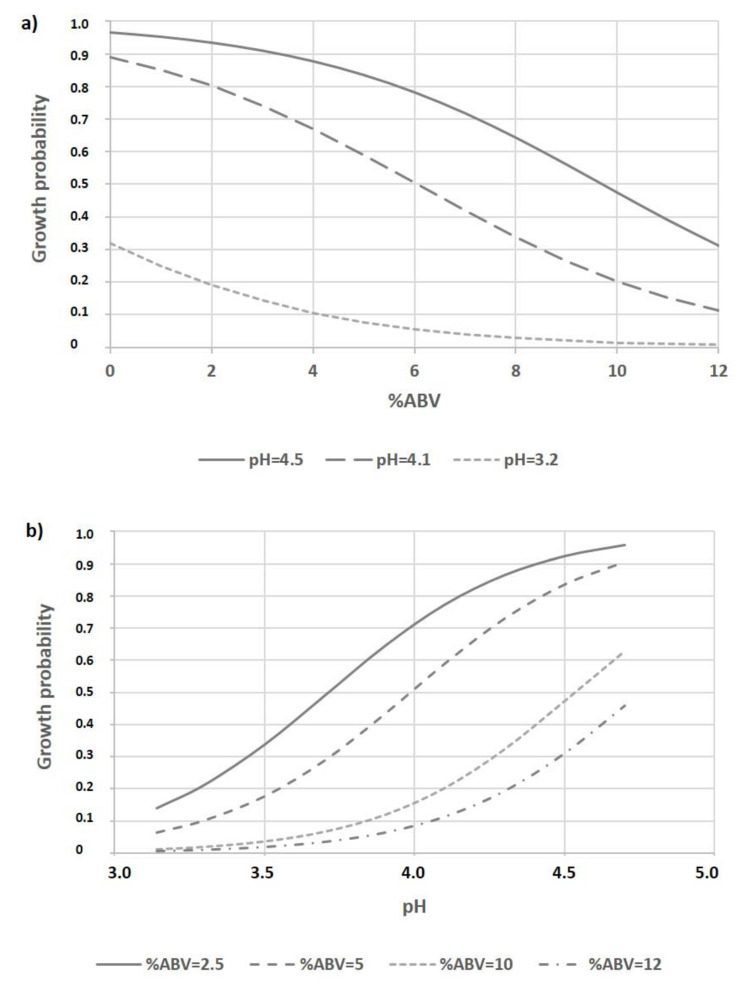
Predicted growth probabilities as a function of: (**a**) % ABV at different pH levels (3.2; 4.1; 4.5); (**b**) pH at different % ABV levels (2.5; 5; 10; 12). Values of % YFE and IBU were set at 2.37 and 30, respectively.

Predicted growth probabilities as a function of % ABV at different pH levels (3.2, 4.1, and 4.5) were depicted for the model in Figure 6a, which showed that at the lowest pH the probability of growth was low, even at the lowest alcohol levels. We found that at higher pH values, higher alcohol concentrations were necessary to produce a strong decrease in the probability of growth. Regarding the influence of pH on the probability of growth at different % ABV levels (2.5, 5, 10, and 12), small transitions of pH values had strong effects on the probability of growth (Figure 6b). Considering that these parameters are usually monitored in craft brewing, the growth of spoilage microorganisms can be controlled mainly by lowering the beer pH, and to a lesser extent by increasing the % ABV. Moreover, IBU can be considered a slighter influencing factor since many beer spoilage microorganisms are hop resistant.

Although each beer has a complex matrix and a set of different strains were used as inoculum under a vast range of four physicochemical parameters (pH, % ABV, IBU, and % YFE), the model correctly predicted as a positive case when microbial growth was observed in beer. This is an important criterion from a preventive perspective to food safety and quality.

### 3.4. Model Validation

External test set was used because it is an effective way to evaluate the predictive ability of a model [40] and to find accurate model that predict data as closely as possible. Data of G/NG observed percentage and predicted probabilities for each of the 10 additional commercial craft beers tested in external validation, and beer classification are available in Supplementary Table S2. The model correctly predicted 100% of the new evaluated cases (Table 4).

In consequence, the model provides the potential to predict craft beer susceptibility to microbial spoilage based on its performance and inherent ability to classify and predict response values both inside and outside the fitted model [29]. The logistic regression model, having just three parameters (pH, % ABV, and IBU), can properly describe the G/NG boundary of spoilage microorganisms in craft beers, thus being easy to use and implement by brewers to accurately predict adequate beers’ formulations. The finding that low pH values had a huge inhibitory impact is also consistent with previous studies based on statistical analysis or regression models. In this way, Fernandez and Simpson [41] described that a beer pH decrease significantly affects the LAB growth, and Uljas et al. [18] concluded that the pH was the most important factor to reduce *E. coli* populations in apple cider. In addition, three different models determined that pH value was statistically significant in cold-filled ready to drink beverages when the spoilage with molds, yeasts, or bacteria were studied [16,17,42]. 

In relation to food safety and quality assurance, this tool provides a good first step for brewers to adjust certain physicochemical parameters to enhance microbial stability, establishing action in the hazard analysis and critical control point plan to reduce the cost and labor time involved in microbial challenge testing. Similarly, this G/NG model might prevent secondary contaminations inside artisan breweries because craft beers are not subjected to pasteurization processing; however, a larger validation samples would be required to detect smaller differences in performance [43].

## 4. Conclusions

A binary logistic regression model predicting the growth of spoilage microorganisms in craft beer have been developed and validated. Antimicrobial hurdles like pH, bitterness units, and alcohol content, which are physicochemical parameters easily controlled by craft brewers, were included in the model. The pH value was the most important factor to predict the susceptibility of craft beer to microbial growth, followed by bitterness units and alcohol content which also showed a good capacity. The G/NG model has shown that there is a goodness of fit and accurate prediction since it correctly classified 276 of 331 analyzed cases (83.4%) and accurately its predictive ability was of 100% in an external validation. In addition, this G/GN model allowed determining beer susceptibility to microbial growth with a sensitivity of 87% and a specificity of 71%.

This tool is an appropriate and robust G/NG model, which has potential to ensure microbiological safety in craft breweries, allowing conscious decision-making at a critical time. However, further research should be carried out to evaluate strains impact and additional factors (phenolic compounds, undissociated SO_2_, dissolved CO_2_, and others) and additional spoilage microorganisms in order to obtain a broader domain and improve the proposed predictive model.

## Figures and Tables

**Table 1:** List of potential beer spoilage microorganisms used in this study.

Code	Microorganism	Original Source	Characteristics in Terms of Brewing Microbiology
L1	*Lactobacilllus brevis*	Craft beer	*L. brevis* is the most prevalent beer spoiler causing more than a half of beer reported incidents. These three strains are hop resistant bacteria [22].
D1	*Lactobacilllus brevis*	Craft brewing environment	
216	*Lactobacilllus brevis*	Beer	
F2	*Pediococcus damnosus*	Beer	The most common beer spoiler.
B6	*Lactobacillus paracasei*	Craft brewing environment	*L. paracasei* and *L. plantarum* are species with relatively weak hop resistance [5].
F1	*Lactobacilllus plantarum*	Alcoholic drink	
B2	*Leuconostoc pseudomesenteroides*	Craft brewing environment	Spoilage incidents caused by *Leuconostoc* sp. are rare except for beers with microbiologically weak features [5].
B1	*Leuconostoc citreum*	Craft brewing environment	
H2	*Dekkera bruxellensis*	Lambic beer	*Dekkera* genus is a typical spoilage yeast for beer.

**Table 2:** Values of main physicochemical parameters of 20 commercially beers.

Beer	% ABV	pH	IBU	% YFE
Stout A	4.2 ± 0.0 ^d^	4.27 ± 0.00 ^b^	31 ± 0 ^k^	1.37 ± 0.05 ^fg^
Stout B	9.0 ± 0.0 ^n^	4.37 ± 0.00 ^b^	44 ± 0 ^l^	1.26 ± 0.00 ^e^
Pale ale A	5.0 ± 0.0 ^e^	4.09 ± 0.00 ^b^	23 ± 0 ^h^	1.39 ± 0.05 ^fg^
Pale ale B	5.0 ± 0.0 ^e^	4.06 ± 0.00 ^b^	22 ± 0 ^g^	2.97 ± 0.05 ^k^
Porter A	5.9 ± 0.0 ^i^	4.08 ± 0.00 ^b^	22 ± 0 ^g^	1.31 ± 0.09 ^ef^
Porter B	5.0 ± 0.0 ^e^	4.14 ± 0.01 ^b^	29 ± 0 ^j^	0.18 ± 0.05 ^a^
Brown ale A	4.3 ± 0.0 ^q^	4.17 ± 0.01 ^b^	21 ± 0 ^f^	0.74 ± 0.05 ^c^
Brown ale B	5.2 ± 0.0 ^f^	4.46 ± 0.00 ^c^	44 ± 0 ^m^	1.45 ± 0.05 ^g^
Amber ale A	5.5 ± 0.0 ^g^	4.25 ± 0.01 ^b^	24 ± 0 ^h^	0.55 ± 0.08 ^b^
Amber ale B	5.8 ± 0.0 ^h^	4.26 ± 0.01 ^b^	31 ± 0 ^k^	0.71 ± 0.00 ^c^
IPA A	7.2 ± 0.0 ^l^	4.32 ± 0.01 ^b^	74 ± 0 ^n^	1.73 ± 0.01 ^h^
IPA B	8.5 ± 0.0 ^m^	4.70 ± 0.02 ^d^	75 ± 0 ^o^	0.92 ± 0.05 ^d^
Strong ale A	6.5 ± 0.0 ^j^	4.20 ± 0.02 ^b^	16 ± 0 ^e^	2.65 ± 0.04 ^j^
Strong ale B	10.0 ± 0.0 ^o^	4.44 ± 0.01 ^c^	25 ± 0 ^i^	1.73 ± 0.00 ^h^
No-alcohol A	0.3 ± 0.0 ^a^	4.20 ± 0.02 ^b^	11 ± 0 ^b^	2.37 ± 0.00 ^i^
No-alcohol B	0.5 ± 0.0 ^b^	4.49 ± 0.00 ^c^	16 ± 0 ^e^	3.05 ± 0.05 ^k^
Wheat beer A	5.5±0.0 ^g^	4.29 ± 0.01 ^b^	15 ± 0 ^d^	1.00 ± 0.05 ^d^
Wheat beer B	3.8 ± 0.0 ^c^	3.14 ± 0.03 ^a^	12 ± 0^c^	2.37 ± 0.00 ^i^
Bock A	12.0 ± 0.0 ^p^	4.66 ± 0.15 ^d^	25 ± 0^i^	1.68 ± 0.12 ^h^
Bock B	7.0 ± 0.0^l^	4.23 ± 0.01 ^b^	10 ± 0^a^	0.76 ± 0.05 ^c^

Values represent means of three different beers ± SD. Values in the same column followed by different letters are significantly different by LSD test (*p* < 0.05).

**Table 3:** Modeling results and variables/coefficients values included in the model.

Nagelkerke R^2^	HL Goodness-of-Fit Test	% Correctly Predicted in Classification Table	Parameter	Coefficients	Standard Error	Wald	Statistical Significance	Odds Ratio	Sensitivity	Specificity
0.41	0.133	83.4%	Constant	−9.608	2.030	22.397	0.000	0.000	87%	71%
			% ABV	−0.346	0.049	50.429	0.000	0.708		
			IBU	−0.042	0.010	16.834	0.000	0.959		
			pH	3.161	0.540	34.306	0.000	23.58		

**Table 4:** Classification Table of the observed and predicted probabilities of the model.

Observed	Predicted		
	Value		% Correct
	Not Easy to Spoil	Easy to Spoil	
Not easy to spoil	1	0	100
Easy to spoil	0	9	100
% Global			100

## Data Availability

The data presented in this study are available on request from the corresponding author.

## Supplementary Materials

The following are available online at https://www.mdpi.com/article/10.3390/foods10081926/s1, Table S1: Data for the Model Development, Table S2: Data for External Model Validation and the observed and predicted probability.
