# Supplementary material for: A Binary Logistic Regression Model as a Tool to Predict Craft Beer Susceptibility to Microbial Spoilage
Source: Foods. 2021 Aug 19;10(8):1926. doi: 10.3390/foods10081926 (PMC8391359; doi:10.3390/foods10081926)
Supplement: Supplementary file 1 [file foods-10-01926-s001.zip › foods-1321810-supplementary.pdf]

A Binary logistic regression model as a tool to predict craft beer susceptibility to microbial spoilage

M. Rodríguez-Saavedra, K. Pérez-Revelo, A. Valero; M. V. Moreno-Arribas, D. González de Llano

Supplementary Table S1: Data for the Model Development

Nº assay	Replica	Hurdle/Beer	% ABV	IBU	pH	% YFE	Strain	G/NG
1	1	%ABV	0	11	4.2	2.37	B1	**1**
	2	%ABV	0	11	4.2	2.37	B1	**1**
	3	%ABV	0	11	4.2	2.37	B1	**1**
2	1	%ABV	2.5	11	4.2	2.37	B1	**1**
	2	%ABV	2.5	11	4.2	2.37	B1	**1**
	3	%ABV	2.5	11	4.2	2.37	B1	**1**
3	1	%ABV	5	11	4.2	2.37	B1	**1**
	2	%ABV	5	11	4.2	2.37	B1	**1**
	3	%ABV	5	11	4.2	2.37	B1	**1**
4	1	%ABV	7.5	11	4.2	2.37	B1	**0**
	2	%ABV	7.5	11	4.2	2.37	B1	**0**
	3	%ABV	7.5	11	4.2	2.37	B1	**0**
5	1	%ABV	10	11	4.2	2.37	B1	**0**
	2	%ABV	10	11	4.2	2.37	B1	**0**
	3	%ABV	10	11	4.2	2.37	B1	**0**
6	1	%ABV	12	11	4.2	2.37	B1	**0**
	2	%ABV	12	11	4.2	2.37	B1	**0**
	3	%ABV	12	11	4.2	2.37	B1	**0**
7	1	% ABV	0	11	4.2	2.37	B2	**1**
	2	%ABV	0	11	4.2	2.37	B2	**1**
	3	%ABV	0	11	4.2	2.37	B2	**1**
8	1	%ABV	2.5	11	4.2	2.37	B2	**1**
	2	%ABV	2.5	11	4.2	2.37	B2	**1**
	3	%ABV	2.5	11	4.2	2.37	B2	**1**
9	1	%ABV	5	11	4.2	2.37	B2	**1**
	2	%ABV	5	11	4.2	2.37	B2	**1**
	3	%ABV	5	11	4.2	2.37	B2	**1**
10	1	%ABV	7.5	11	4.2	2.37	B2	**0**
	2	%ABV	7.5	11	4.2	2.37	B2	**0**
	3	%ABV	7.5	11	4.2	2.37	B2	**0**
11	1	%ABV	10	11	4.2	2.37	B2	**0**
	2	%ABV	10	11	4.2	2.37	B2	**0**
	3	%ABV	10	11	4.2	2.37	B2	**0**
12	1	%ABV	12	11	4.2	2.37	B2	**0**
	2	%ABV	12	11	4.2	2.37	B2	**0**
	3	%ABV	12	11	4.2	2.37	B2	**0**
13	1	%ABV	0	11	4.2	2.37	F2	**1**
	2	%ABV	0	11	4.2	2.37	F2	**1**
	3	%ABV	0	11	4.2	2.37	F2	**1**
14	1	%ABV	2.5	11	4.2	2.37	F2	**1**
	2	%ABV	2.5	11	4.2	2.37	F2	**1**
	3	%ABV	2.5	11	4.2	2.37	F2	**1**
15	1	%ABV	5	11	4.2	2.37	F2	**1**
	2	%ABV	5	11	4.2	2.37	F2	**1**
	3	%ABV	5	11	4.2	2.37	F2	**0**
16	1	%ABV	10	11	4.2	2.37	F2	**0**
	2	%ABV	10	11	4.2	2.37	F2	**0**
	3	%ABV	10	11	4.2	2.37	F2	**0**
17	1	%ABV	12	11	4.2	2.37	F2	**0**
	2	%ABV	12	11	4.2	2.37	F2	**0**
	3	%ABV	12	11	4.2	2.37	F2	**0**
18	1	%ABV	0	11	4.2	2.37	F1	**1**
	2	%ABV	0	11	4.2	2.37	F1	**1**
	3	%ABV	0	11	4.2	2.37	F1	**1**
19	1	%ABV	2.5	11	4.2	2.37	F1	**1**
	2	%ABV	2.5	11	4.2	2.37	F1	**1**
	3	%ABV	2.5	11	4.2	2.37	F1	**1**
20	1	%ABV	5	11	4.2	2.37	F1	**1**
	2	%ABV	5	11	4.2	2.37	F1	**1**
	3	%ABV	5	11	4.2	2.37	F1	**1**
21	1	%ABV	7.5	11	4.2	2.37	F1	**0**
	2	%ABV	7.5	11	4.2	2.37	F1	**0**

	3	%ABV	7.5	11	4.2	2.37	F1	**0**
22	1	%ABV	10	11	4.2	2.37	F1	**0**
	2	%ABV	10	11	4.2	2.37	F1	**0**
	3	%ABV	10	11	4.2	2.37	F1	**0**
23	1	%ABV	12	11	4.2	2.37	F1	**0**
	2	%ABV	12	11	4.2	2.37	F2	**0**
	3	%ABV	12	11	4.2	2.37	F2	**0**
24	1	%ABV	0	11	4.2	2.37	B6	**1**
	2	%ABV	0	11	4.2	2.37	B6	**1**
	3	%ABV	0	11	4.2	2.37	B6	**1**
25	1	%ABV	2.5	11	4.2	2.37	B6	**1**
	2	%ABV	2.5	11	4.2	2.37	B6	**1**
	3	%ABV	2.5	11	4.2	2.37	B6	**1**
26	1	%ABV	5	11	4.2	2.37	B6	**1**
	2	%ABV	5	11	4.2	2.37	B6	**1**
	3	%ABV	5	11	4.2	2.37	B6	**1**
27	1	%ABV	7.5	11	4.2	2.37	B6	**0**
	2	%ABV	7.5	11	4.2	2.37	B6	**0**
	3	%ABV	7.5	11	4.2	2.37	B6	**0**
28	1	%ABV	10	11	4.2	2.37	B6	**0**
	2	%ABV	10	11	4.2	2.37	B6	**0**
	3	%ABV	10	11	4.2	2.37	B6	**0**
29	1	%ABV	12	11	4.2	2.37	B6	**0**
	2	%ABV	12	11	4.2	2.37	B6	**0**
	3	%ABV	12	11	4.2	2.37	B6	**0**
30	1	%ABV	0	11	4.2	2.37	216	**1**
	2	%ABV	0	11	4.2	2.37	216	**1**
	3	%ABV	0	11	4.2	2.37	216	**1**
31	1	%ABV	2.5	11	4.2	2.37	216	**1**
	2	%ABV	2.5	11	4.2	2.37	216	**1**
	3	%ABV	2.5	11	4.2	2.37	216	**1**
32	1	%ABV	5	11	4.2	2.37	216	**1**
	2	%ABV	5	11	4.2	2.37	216	**1**
	3	%ABV	5	11	4.2	2.37	216	**1**
33	1	%ABV	7.5	11	4.2	2.37	216	**1**
	2	%ABV	7.5	11	4.2	2.37	216	**1**
	3	%ABV	7.5	11	4.2	2.37	216	**1**
34	1	%ABV	10	11	4.2	2.37	216	**1**
	2	%ABV	10	11	4.2	2.37	216	**1**
	3	%ABV	10	11	4.2	2.37	216	**1**
35	1	%ABV	12	11	4.2	2.37	216	**0**
	2	%ABV	12	11	4.2	2.37	216	**0**
	3	%ABV	12	11	4.2	2.37	216	**0**
36	1	%ABV	0	11	4.2	2.37	D1	**1**
	2	%ABV	0	11	4.2	2.37	D1	**1**
	3	%ABV	0	11	4.2	2.37	D1	**1**
37	1	%ABV	2.5	11	4.2	2.37	D1	**1**
	2	%ABV	2.5	11	4.2	2.37	D1	**1**
	3	%ABV	2.5	11	4.2	2.37	D1	**1**
38	1	%ABV	5	11	4.2	2.37	D1	**1**
	2	%ABV	5	11	4.2	2.37	D1	**1**
	3	%ABV	5	11	4.2	2.37	D1	**1**
39	1	%ABV	7.5	11	4.2	2.37	D1	**1**
	2	%ABV	7.5	11	4.2	2.37	D1	**1**
	3	%ABV	7.5	11	4.2	2.37	D1	**1**
40	1	%ABV	10	11	4.2	2.37	D1	**1**
	2	%ABV	10	11	4.2	2.37	D1	**1**
	3	%ABV	10	11	4.2	2.37	D1	**1**
41	1	%ABV	12	11	4.2	2.37	D1	**0**
	2	%ABV	12	11	4.2	2.37	D1	**0**
	3	%ABV	12	11	4.2	2.37	D1	**0**
42	1	%ABV	0	11	4.2	2.37	L1	**1**
	2	%ABV	0	11	4.2	2.37	L1	**1**
	3	%ABV	0	11	4.2	2.37	L1	**1**
43	1	%ABV	2.5	11	4.2	2.37	L1	**1**
	2	%ABV	2.5	11	4.2	2.37	L1	**1**
	3	%ABV	2.5	11	4.2	2.37	L1	**1**
44	1	%ABV	5	11	4.2	2.37	L1	**1**

	2	%ABV	5	11	4.2	2.37	L1	**1**
	3	%ABV	5	11	4.2	2.37	L1	**1**
45	1	%ABV	7.5	11	4.2	2.37	L1	**1**
	2	%ABV	7.5	11	4.2	2.37	L1	**1**
	3	%ABV	7.5	11	4.2	2.37	L1	**1**
46	1	%ABV	10	11	4.2	2.37	L1	**1**
	2	%ABV	10	11	4.2	2.37	L1	**1**
	3	%ABV	10	11	4.2	2.37	L1	**1**
47	1	%ABV	12	11	4.2	2.37	L1	**0**
	2	%ABV	12	11	4.2	2.37	L1	**0**
	3	%ABV	12	11	4.2	2.37	L1	**0**
48	1	%ABV	0	11	4.2	2.37	H2	**1**
	2	%ABV	0	11	4.2	2.37	H2	**1**
	3	%ABV	0	11	4.2	2.37	H2	**1**
49	1	%ABV	2.5	11	4.2	2.37	H2	**1**
	2	%ABV	2.5	11	4.2	2.37	H2	**1**
	3	%ABV	2.5	11	4.2	2.37	H2	**1**
50	1	%ABV	5	11	4.2	2.37	H2	**1**
	2	%ABV	5	11	4.2	2.37	H2	**1**
	3	%ABV	5	11	4.2	2.37	H2	**1**
51	1	%ABV	7.5	11	4.2	2.37	H2	**1**
	2	%ABV	7.5	11	4.2	2.37	H2	**1**
	3	%ABV	7.5	11	4.2	2.37	H2	**1**
52	1	%ABV	10	11	4.2	2.37	H2	**1**
	2	%ABV	10	11	4.2	2.37	H2	**1**
	3	%ABV	10	11	4.2	2.37	H2	**1**
53	1	%ABV	12	11	4.2	2.37	H2	**1**
	2	%ABV	12	11	4.2	2.37	H2	**1**
	3	%ABV	12	11	4.2	2.37	H2	**1**
54	1	IBU	0.1	10	4.2	2	B1	**1**
	2	IBU	0.1	10	4.2	2	B1	**1**
	3	IBU	0.1	10	4.2	2	B1	**1**
55	1	IBU	0.1	15	4.3	2.2	B1	**1**
	2	IBU	0.1	15	4.3	2.2	B1	**1**
	3	IBU	0.1	15	4.3	2.2	B1	**1**
56	1	IBU	0.1	20	4.4	2.4	B1	**1**
	2	IBU	0.1	20	4.4	2.4	B1	**1**
	3	IBU	0.1	20	4.4	2.4	B1	**1**
57	1	IBU	0.1	30	4.5	2.6	B1	**1**
	2	IBU	0.1	30	4.5	2.6	B1	**1**
	3	IBU	0.1	30	4.5	2.6	B1	**1**
58	1	IBU	0.1	40	4.55	2.8	B1	**0**
	2	IBU	0.1	40	4.55	2.8	B1	**0**
	3	IBU	0.1	40	4.55	2.8	B1	**0**
59	1	IBU	0.1	50	4.6	3	B1	**0**
	2	IBU	0.1	50	4.6	3	B1	**0**
	3	IBU	0.1	50	4.6	3	B1	**0**
60	1	IBU	0.1	10	4.2	2	B2	**1**
	2	IBU	0.1	10	4.2	2	B2	**1**
	3	IBU	0.1	10	4.2	2	B2	**1**
61	1	IBU	0.1	15	4.3	2.2	B2	**1**
	2	IBU	0.1	15	4.3	2.2	B2	**1**
	3	IBU	0.1	15	4.3	2.2	B2	**1**
62	1	IBU	0.1	20	4.4	2.4	B2	**1**
	2	IBU	0.1	20	4.4	2.4	B2	**1**
	3	IBU	0.1	20	4.4	2.4	B2	**1**
63	1	IBU	0.1	30	4.5	2.6	B2	**1**
	2	IBU	0.1	30	4.5	2.6	B2	**1**
	3	IBU	0.1	30	4.5	2.6	B2	**1**
64	1	IBU	0.1	40	4.55	2.8	B2	**0**
	2	IBU	0.1	40	4.55	2.8	B2	**0**
	3	IBU	0.1	40	4.55	2.8	B2	**0**
65	1	IBU	0.1	50	4.6	3	B2	**0**
	2	IBU	0.1	50	4.6	3	B2	**0**
	3	IBU	0.1	50	4.6	3	B2	**0**
66	1	IBU	0.1	10	4.2	2	F2	**1**
	2	IBU	0.1	10	4.2	2	F2	**1**
	3	IBU	0.1	10	4.2	2	F2	**1**

67	1	IBU	0.1	15	4.3	2.2	F2	**1**
	2	IBU	0.1	15	4.3	2.2	F2	**1**
	3	IBU	0.1	15	4.3	2.2	F2	**1**
68	1	IBU	0.1	20	4.4	2.4	F2	**1**
	2	IBU	0.1	20	4.4	2.4	F2	**1**
	3	IBU	0.1	20	4.4	2.4	F2	**1**
69	1	IBU	0.1	30	4.5	2.6	F2	**1**
	2	IBU	0.1	30	4.5	2.6	F2	**1**
	3	IBU	0.1	30	4.5	2.6	F2	**1**
70	1	IBU	0.1	40	4.55	2.8	F2	**1**
	2	IBU	0.1	40	4.55	2.8	F2	**1**
	3	IBU	0.1	40	4.55	2.8	F2	**1**
71	1	IBU	0.1	50	4.6	3	F2	**1**
	2	IBU	0.1	50	4.6	3	F2	**1**
	3	IBU	0.1	50	4.6	3	F2	**1**
72	1	IBU	0.1	10	4.2	2	F1	**1**
	2	IBU	0.1	10	4.2	2	F1	**1**
	3	IBU	0.1	10	4.2	2	F1	**1**
73	1	IBU	0.1	15	4.3	2.2	F1	**1**
	2	IBU	0.1	15	4.3	2.2	F1	**1**
	3	IBU	0.1	15	4.3	2.2	F1	**1**
74	1	IBU	0.1	20	4.4	2.4	F1	**1**
	2	IBU	0.1	20	4.4	2.4	F1	**1**
	3	IBU	0.1	20	4.4	2.4	F1	**1**
75	1	IBU	0.1	30	4.5	2.6	F1	**1**
	2	IBU	0.1	30	4.5	2.6	F1	**1**
	3	IBU	0.1	30	4.5	2.6	F1	**1**
76	1	IBU	0.1	40	4.55	2.8	F1	**0**
	2	IBU	0.1	40	4.55	2.8	F1	**0**
	3	IBU	0.1	40	4.55	2.8	F1	**0**
77	1	IBU	0.1	50	4.6	3	F1	**0**
	2	IBU	0.1	50	4.6	3	F1	**0**
	3	IBU	0.1	50	4.6	3	F1	**0**
78	1	IBU	0.1	10	4.2	2	B6	**1**
	2	IBU	0.1	10	4.2	2	B6	**1**
	3	IBU	0.1	10	4.2	2	B6	**1**
79	1	IBU	0.1	15	4.3	2.2	B6	**1**
	2	IBU	0.1	15	4.3	2.2	B6	**1**
	3	IBU	0.1	15	4.3	2.2	B6	**1**
80	1	IBU	0.1	20	4.4	2.4	B6	**1**
	2	IBU	0.1	20	4.4	2.4	B6	**1**
	3	IBU	0.1	20	4.4	2.4	B6	**1**
81	1	IBU	0.1	30	4.5	2.6	B6	**1**
	2	IBU	0.1	30	4.5	2.6	B6	**1**
	3	IBU	0.1	30	4.5	2.6	B6	**1**
82	1	IBU	0.1	40	4.55	2.8	B6	**1**
	2	IBU	0.1	40	4.55	2.8	B6	**1**
	3	IBU	0.1	40	4.55	2.8	B6	**1**
83	1	IBU	0.1	50	4.6	3	B6	**1**
	2	IBU	0.1	50	4.6	3	B6	**1**
	3	IBU	0.1	50	4.6	3	B6	**0**
84	1	IBU	0.1	10	4.2	2	216	**1**
	2	IBU	0.1	10	4.2	2	216	**1**
	3	IBU	0.1	10	4.2	2	216	**1**
85	1	IBU	0.1	15	4.3	2.2	216	**1**
	2	IBU	0.1	15	4.3	2.2	216	**1**
	3	IBU	0.1	15	4.3	2.2	216	**1**
86	1	IBU	0.1	20	4.4	2.4	216	**1**
	2	IBU	0.1	20	4.4	2.4	216	**1**
	3	IBU	0.1	20	4.4	2.4	216	**1**
87	1	IBU	0.1	30	4.5	2.6	216	**1**
	2	IBU	0.1	30	4.5	2.6	216	**1**
	3	IBU	0.1	30	4.5	2.6	216	**1**
88	1	IBU	0.1	40	4.55	2.8	216	**1**
	2	IBU	0.1	40	4.55	2.8	216	**1**
	3	IBU	0.1	40	4.55	2.8	216	**1**
89	1	IBU	0.1	50	4.6	3	216	**1**
	2	IBU	0.1	50	4.6	3	216	**1**

	3	IBU	0.1	50	4.6	3	216	**1**
90	1	IBU	0.1	10	4.2	2	D1	**1**
	2	IBU	0.1	10	4.2	2	D1	**1**
	3	IBU	0.1	10	4.2	2	D1	**1**
91	1	IBU	0.1	15	4.3	2.2	D1	**1**
	2	IBU	0.1	15	4.3	2.2	D1	**1**
	3	IBU	0.1	15	4.3	2.2	D1	**1**
92	1	IBU	0.1	20	4.4	2.4	D1	**1**
	2	IBU	0.1	20	4.4	2.4	D1	**1**
	3	IBU	0.1	20	4.4	2.4	D1	**1**
93	1	IBU	0.1	30	4.5	2.6	D1	**1**
	2	IBU	0.1	30	4.5	2.6	D1	**1**
	3	IBU	0.1	30	4.5	2.6	D1	**1**
94	1	IBU	0.1	40	4.55	2.8	D1	**1**
	2	IBU	0.1	40	4.55	2.8	D1	**1**
	3	IBU	0.1	40	4.55	2.8	D1	**1**
95	1	IBU	0.1	50	4.6	3	D1	**1**
	2	IBU	0.1	50	4.6	3	D1	**1**
	3	IBU	0.1	50	4.6	3	D1	**1**
96	1	IBU	0.1	10	4.2	2	L1	**1**
	2	IBU	0.1	10	4.2	2	L1	**1**
	3	IBU	0.1	10	4.2	2	L1	**1**
97	1	IBU	0.1	15	4.3	2.2	L1	**1**
	2	IBU	0.1	15	4.3	2.2	L1	**1**
	3	IBU	0.1	15	4.3	2.2	L1	**1**
98	1	IBU	0.1	20	4.4	2.4	L1	**1**
	2	IBU	0.1	20	4.4	2.4	L1	**1**
	3	IBU	0.1	20	4.4	2.4	L1	**1**
99	1	IBU	0.1	30	4.5	2.6	L1	**1**
	2	IBU	0.1	30	4.5	2.6	L1	**1**
	3	IBU	0.1	30	4.5	2.6	L1	**1**
100	1	IBU	0.1	40	4.55	2.8	L1	**1**
	2	IBU	0.1	40	4.55	2.8	L1	**1**
	3	IBU	0.1	40	4.55	2.8	L1	**1**
101	1	IBU	0.1	50	4.6	3	L1	**1**
	2	IBU	0.1	50	4.6	3	L1	**1**
	3	IBU	0.1	50	4.6	3	L1	**1**
102	1	IBU	0.1	10	4.2	2	H2	**1**
	2	IBU	0.1	10	4.2	2	H2	**1**
	3	IBU	0.1	10	4.2	2	H2	**1**
103	1	IBU	0.1	15	4.3	2.2	H2	**1**
	2	IBU	0.1	15	4.3	2.2	H2	**1**
	3	IBU	0.1	15	4.3	2.2	H2	**1**
104	1	IBU	0.1	20	4.4	2.4	H2	**1**
	2	IBU	0.1	20	4.4	2.4	H2	**1**
	3	IBU	0.1	20	4.4	2.4	H2	**1**
105	1	IBU	0.1	30	4.5	2.6	H2	**1**
	2	IBU	0.1	30	4.5	2.6	H2	**1**
	3	IBU	0.1	30	4.5	2.6	H2	**1**
106	1	IBU	0.1	40	4.55	2.8	H2	**1**
	2	IBU	0.1	40	4.55	2.8	H2	**1**
	3	IBU	0.1	40	4.55	2.8	H2	**1**
107	1	IBU	0.1	50	4.6	3	H2	**1**
	2	IBU	0.1	50	4.6	3	H2	**1**
	3	IBU	0.1	50	4.6	3	H2	**1**
108	1	pH	0.5	11	3.2	1.45	B1	**0**
	2	pH	0.5	11	3.2	1.45	B1	**0**
	3	pH	0.5	11	3.2	1.45	B1	**0**
109	1	pH	0.5	11	3.5	1.45	B1	**0**
	2	pH	0.5	11	3.5	1.45	B1	**0**
	3	pH	0.5	11	3.5	1.45	B1	**0**
110	1	pH	0.5	11	3.8	1.45	B1	**1**
	2	pH	0.5	11	3.8	1.45	B1	**1**
	3	pH	0.5	11	3.8	1.45	B1	**0**
111	1	pH	0.5	11	4.1	1.45	B1	**1**
	2	pH	0.5	11	4.1	1.45	B1	**1**
	3	pH	0.5	11	4.1	1.45	B1	**1**
112	1	pH	0.5	11	4.4	1.45	B1	**1**

	2	pH	0.5	11	4.4	1.45	B1	**1**
	3	pH	0.5	11	4.4	1.45	B1	**1**
113	1	pH	0.5	11	4.7	1.45	B1	**1**
	2	pH	0.5	11	4.7	1.45	B1	**1**
	3	pH	0.5	11	4.7	1.45	B1	**1**
114	1	pH	0.5	11	3.2	1.45	B2	**0**
	2	pH	0.5	11	3.2	1.45	B2	**0**
	3	pH	0.5	11	3.2	1.45	B2	**0**
115	1	pH	0.5	11	3.5	1.45	B2	**1**
	2	pH	0.5	11	3.5	1.45	B2	**1**
	3	pH	0.5	11	3.5	1.45	B2	**0**
116	1	pH	0.5	11	3.8	1.45	B2	**1**
	2	pH	0.5	11	3.8	1.45	B2	**1**
	3	pH	0.5	11	3.8	1.45	B2	**1**
117	1	pH	0.5	11	4.1	1.45	B2	**1**
	2	pH	0.5	11	4.1	1.45	B2	**1**
	3	pH	0.5	11	4.1	1.45	B2	**1**
118	1	pH	0.5	11	4.4	1.45	B2	**1**
	2	pH	0.5	11	4.4	1.45	B2	**1**
	3	pH	0.5	11	4.4	1.45	B2	**1**
119	1	pH	0.5	11	4.7	1.45	B2	**1**
	2	pH	0.5	11	4.7	1.45	B2	**1**
	3	pH	0.5	11	4.7	1.45	B2	**1**
120	1	pH	0.5	11	3.2	1.45	F2	**0**
	2	pH	0.5	11	3.2	1.45	F2	**0**
	3	pH	0.5	11	3.2	1.45	F2	**0**
121	1	pH	0.5	11	3.5	1.45	F2	**1**
	2	pH	0.5	11	3.5	1.45	F2	**1**
	3	pH	0.5	11	3.5	1.45	F2	**0**
122	1	pH	0.5	11	3.8	1.45	F2	**1**
	2	pH	0.5	11	3.8	1.45	F2	**1**
	3	pH	0.5	11	3.8	1.45	F2	**1**
123	1	pH	0.5	11	4.1	1.45	F2	**1**
	2	pH	0.5	11	4.1	1.45	F2	**1**
	3	pH	0.5	11	4.1	1.45	F2	**1**
124	1	pH	0.5	11	4.4	1.45	F2	**1**
	2	pH	0.5	11	4.4	1.45	F2	**1**
	3	pH	0.5	11	4.4	1.45	F2	**1**
125	1	pH	0.5	11	4.7	1.45	F2	**1**
	2	pH	0.5	11	4.7	1.45	F2	**1**
	3	pH	0.5	11	4.7	1.45	F2	**1**
126	1	pH	0.5	11	3.2	1.45	F1	**0**
	2	pH	0.5	11	3.2	1.45	F1	**0**
	3	pH	0.5	11	3.2	1.45	F1	**0**
127	1	pH	0.5	11	3.5	1.45	F1	**1**
	2	pH	0.5	11	3.5	1.45	F1	**1**
	3	pH	0.5	11	3.5	1.45	F1	**0**
128	1	pH	0.5	11	3.8	1.45	F1	**1**
	2	pH	0.5	11	3.8	1.45	F1	**1**
	3	pH	0.5	11	3.8	1.45	F1	**1**
129	1	pH	0.5	11	4.1	1.45	F1	**1**
	2	pH	0.5	11	4.1	1.45	F1	**1**
	3	pH	0.5	11	4.1	1.45	F1	**1**
130	1	pH	0.5	11	4.4	1.45	F1	**1**
	2	pH	0.5	11	4.4	1.45	F1	**1**
	3	pH	0.5	11	4.4	1.45	F1	**1**
131	1	pH	0.5	11	4.7	1.45	F1	**1**
	2	pH	0.5	11	4.7	1.45	F1	**1**
	3	pH	0.5	11	4.7	1.45	F1	**1**
132	1	pH	0.5	11	3.2	1.45	B6	**0**
	2	pH	0.5	11	3.2	1.45	B6	**0**
	3	pH	0.5	11	3.2	1.45	B6	**0**
133	1	pH	0.5	11	3.5	1.45	B6	**0**
	2	pH	0.5	11	3.5	1.45	B6	**0**
	3	pH	0.5	11	3.5	1.45	B6	**0**
134	1	pH	0.5	11	3.8	1.45	B6	**0**
	2	pH	0.5	11	3.8	1.45	B6	**0**
	3	pH	0.5	11	3.8	1.45	B6	**0**

135	1	pH	0.5	11	4.1	1.45	B6	**1**
	2	pH	0.5	11	4.1	1.45	B6	**1**
	3	pH	0.5	11	4.1	1.45	B6	**1**
136	1	pH	0.5	11	4.4	1.45	B6	**1**
	2	pH	0.5	11	4.4	1.45	B6	**1**
	3	pH	0.5	11	4.4	1.45	B6	**1**
137	1	pH	0.5	11	4.7	1.45	B6	**1**
	2	pH	0.5	11	4.7	1.45	B6	**1**
	3	pH	0.5	11	4.7	1.45	B6	**1**
138	1	pH	0.5	11	3.2	1.45	216	**0**
	2	pH	0.5	11	3.2	1.45	216	**0**
	3	pH	0.5	11	3.2	1.45	216	**0**
139	1	pH	0.5	11	3.5	1.45	216	**1**
	2	pH	0.5	11	3.5	1.45	216	**1**
	3	pH	0.5	11	3.5	1.45	216	**0**
140	1	pH	0.5	11	3.8	1.45	216	**1**
	2	pH	0.5	11	3.8	1.45	216	**1**
	3	pH	0.5	11	3.8	1.45	216	**1**
141	1	pH	0.5	11	4.1	1.45	216	**1**
	2	pH	0.5	11	4.1	1.45	216	**1**
	3	pH	0.5	11	4.1	1.45	216	**1**
142	1	pH	0.5	11	4.4	1.45	216	**1**
	2	pH	0.5	11	4.4	1.45	216	**1**
	3	pH	0.5	11	4.4	1.45	216	**1**
143	1	pH	0.5	11	4.7	1.45	216	**1**
	2	pH	0.5	11	4.7	1.45	216	**1**
	3	pH	0.5	11	4.7	1.45	216	**1**
144	1	pH	0.5	11	3.2	1.45	D1	**0**
	2	pH	0.5	11	3.2	1.45	D1	**0**
	3	pH	0.5	11	3.2	1.45	D1	**0**
145	1	pH	0.5	11	3.5	1.45	D1	**1**
	2	pH	0.5	11	3.5	1.45	D1	**1**
	3	pH	0.5	11	3.5	1.45	D1	**0**
146	1	pH	0.5	11	3.8	1.45	D1	**1**
	2	pH	0.5	11	3.8	1.45	D1	**1**
	3	pH	0.5	11	3.8	1.45	D1	**1**
147	1	pH	0.5	11	4.1	1.45	D1	**1**
	2	pH	0.5	11	4.1	1.45	D1	**1**
	3	pH	0.5	11	4.1	1.45	D1	**1**
148	1	pH	0.5	11	4.4	1.45	D1	**1**
	2	pH	0.5	11	4.4	1.45	D1	**1**
	3	pH	0.5	11	4.4	1.45	D1	**1**
149	1	pH	0.5	11	4.7	1.45	D1	**1**
	2	pH	0.5	11	4.7	1.45	D1	**1**
	3	pH	0.5	11	4.7	1.45	D1	**1**
150	1	pH	0.5	11	3.2	1.45	L1	**0**
	2	pH	0.5	11	3.2	1.45	L1	**0**
	3	pH	0.5	11	3.2	1.45	L1	**0**
151	1	pH	0.5	11	3.5	1.45	L1	**0**
	2	pH	0.5	11	3.5	1.45	L1	**0**
	3	pH	0.5	11	3.5	1.45	L1	**0**
152	1	pH	0.5	11	3.8	1.45	L1	**1**
	2	pH	0.5	11	3.8	1.45	L1	**0**
	3	pH	0.5	11	3.8	1.45	L1	**1**
153	1	pH	0.5	11	4.1	1.45	L1	**1**
	2	pH	0.5	11	4.1	1.45	L1	**1**
	3	pH	0.5	11	4.1	1.45	L1	**1**
154	1	pH	0.5	11	4.4	1.45	L1	**1**
	2	pH	0.5	11	4.4	1.45	L1	**1**
	3	pH	0.5	11	4.4	1.45	L1	**1**
155	1	pH	0.5	11	4.7	1.45	L1	**1**
	2	pH	0.5	11	4.7	1.45	L1	**1**
	3	pH	0.5	11	4.7	1.45	L1	**1**
156	1	pH	0.5	11	3.2	1.45	H2	**1**
	2	pH	0.5	11	3.2	1.45	H2	**1**
	3	pH	0.5	11	3.2	1.45	H2	**0**
157	1	pH	0.5	11	3.5	1.45	H2	**1**
	2	pH	0.5	11	3.5	1.45	H2	**1**

	3	pH	0.5	11	3.5	1.45	H2	**1**
158	1	pH	0.5	11	3.8	1.45	H2	**1**
	2	pH	0.5	11	3.8	1.45	H2	**1**
	3	pH	0.5	11	3.8	1.45	H2	**1**
159	1	pH	0.5	11	4.1	1.45	H2	**1**
	2	pH	0.5	11	4.1	1.45	H2	**1**
	3	pH	0.5	11	4.1	1.45	H2	**1**
160	1	pH	0.5	11	4.4	1.45	H2	**1**
	2	pH	0.5	11	4.4	1.45	H2	**1**
	3	pH	0.5	11	4.4	1.45	H2	**1**
161	1	pH	0.5	11	4.7	1.45	H2	**1**
	2	pH	0.5	11	4.7	1.45	H2	**1**
	3	pH	0.5	11	4.7	1.45	H2	**1**
162	1	YFE	0.1	10	4.2	0.76	B1	**1**
	2	YFE	0.1	10	4.2	0.76	B1	**1**
	3	YFE	0.1	10	4.2	0.76	B1	**1**
163	1	YFE	0.1	10	4.2	1.16	B1	**1**
	2	YFE	0.1	10	4.2	1.16	B1	**1**
	3	YFE	0.1	10	4.2	1.16	B1	**1**
164	1	YFE	0.1	10	4.2	1.96	B1	**1**
	2	YFE	0.1	10	4.2	1.96	B1	**1**
	3	YFE	0.1	10	4.2	1.96	B1	**1**
165	1	YFE	0.1	10	4.2	2.36	B1	**1**
	2	YFE	0.1	10	4.2	2.36	B1	**1**
	3	YFE	0.1	10	4.2	2.36	B1	**1**
166	1	YFE	0.1	10	4.2	0.76	B2	**1**
	2	YFE	0.1	10	4.2	0.76	B2	**1**
	3	YFE	0.1	10	4.2	0.76	B2	**1**
167	1	YFE	0.1	10	4.2	1.16	B2	**1**
	2	YFE	0.1	10	4.2	1.16	B2	**1**
	3	YFE	0.1	10	4.2	1.16	B2	**1**
168	1	YFE	0.1	10	4.2	1.96	B2	**1**
	2	YFE	0.1	10	4.2	1.96	B2	**1**
	3	YFE	0.1	10	4.2	1.96	B2	**1**
169	1	YFE	0.1	10	4.2	2.36	B2	**1**
	2	YFE	0.1	10	4.2	2.36	B2	**1**
	3	YFE	0.1	10	4.2	2.36	B2	**1**
170	1	YFE	0.1	10	4.2	0.76	F2	**1**
	2	YFE	0.1	10	4.2	0.76	F2	**1**
	3	YFE	0.1	10	4.2	0.76	F2	**1**
171	1	YFE	0.1	10	4.2	1.16	F2	**1**
	2	YFE	0.1	10	4.2	1.16	F2	**1**
	3	YFE	0.1	10	4.2	1.16	F2	**1**
172	1	YFE	0.1	10	4.2	1.96	F2	**1**
	2	YFE	0.1	10	4.2	1.96	F2	**1**
	3	YFE	0.1	10	4.2	1.96	F2	**1**
173	1	YFE	0.1	10	4.2	2.36	F2	**1**
	2	YFE	0.1	10	4.2	2.36	F2	**1**
	3	YFE	0.1	10	4.2	2.36	F2	**1**
174	1	YFE	0.1	10	4.2	0.76	F1	**1**
	2	YFE	0.1	10	4.2	0.76	F1	**1**
	3	YFE	0.1	10	4.2	0.76	F1	**1**
175	1	YFE	0.1	10	4.2	1.16	F1	**1**
	2	YFE	0.1	10	4.2	1.16	F1	**1**
	3	YFE	0.1	10	4.2	1.16	F1	**1**
176	1	YFE	0.1	10	4.2	1.96	F1	**1**
	2	YFE	0.1	10	4.2	1.96	F1	**1**
	3	YFE	0.1	10	4.2	1.96	F1	**1**
177	1	YFE	0.1	10	4.2	2.36	F1	**1**
	2	YFE	0.1	10	4.2	2.36	F1	**1**
	3	YFE	0.1	10	4.2	2.36	F1	**1**
178	1	YFE	0.1	10	4.2	0.76	B6	**1**
	2	YFE	0.1	10	4.2	0.76	B6	**1**
	3	YFE	0.1	10	4.2	0.76	B6	**1**
179	1	YFE	0.1	10	4.2	1.16	B6	**1**
	2	YFE	0.1	10	4.2	1.16	B6	**1**
	3	YFE	0.1	10	4.2	1.16	B6	**1**
180	1	YFE	0.1	10	4.2	1.96	B6	**1**

	2	YFE	0.1	10	4.2	1.96	B6	**1**
	3	YFE	0.1	10	4.2	1.96	B6	**1**
177	1	YFE	0.1	10	4.2	2.36	B6	**1**
	2	YFE	0.1	10	4.2	2.36	B6	**1**
	3	YFE	0.1	10	4.2	2.36	B6	**1**
182	1	YFE	0.1	10	4.2	0.76	216	**1**
	2	YFE	0.1	10	4.2	0.76	216	**1**
	3	YFE	0.1	10	4.2	0.76	216	**1**
183	1	YFE	0.1	10	4.2	1.16	216	**1**
	2	YFE	0.1	10	4.2	1.16	216	**1**
	3	YFE	0.1	10	4.2	1.16	216	**1**
184	1	YFE	0.1	10	4.2	1.96	216	**1**
	2	YFE	0.1	10	4.2	1.96	216	**1**
	3	YFE	0.1	10	4.2	1.96	216	**1**
185	1	YFE	0.1	10	4.2	2.36	216	**1**
	2	YFE	0.1	10	4.2	2.36	216	**1**
	3	YFE	0.1	10	4.2	2.36	216	**1**
186	1	YFE	0.1	10	4.2	0.76	D1	**1**
	2	YFE	0.1	10	4.2	0.76	D1	**1**
	3	YFE	0.1	10	4.2	0.76	D1	**1**
187	1	YFE	0.1	10	4.2	1.16	D1	**1**
	2	YFE	0.1	10	4.2	1.16	D1	**1**
	3	YFE	0.1	10	4.2	1.16	D1	**1**
188	1	YFE	0.1	10	4.2	1.96	D1	**1**
	2	YFE	0.1	10	4.2	1.96	D1	**1**
	3	YFE	0.1	10	4.2	1.96	D1	**1**
189	1	YFE	0.1	10	4.2	2.36	D1	**1**
	2	YFE	0.1	10	4.2	2.36	D1	**1**
	3	YFE	0.1	10	4.2	2.36	D1	**1**
190	1	YFE	0.1	10	4.2	0.76	L1	**1**
	2	YFE	0.1	10	4.2	0.76	L1	**1**
	3	YFE	0.1	10	4.2	0.76	L1	**1**
191	1	YFE	0.1	10	4.2	1.16	L1	**1**
	2	YFE	0.1	10	4.2	1.16	L1	**1**
	3	YFE	0.1	10	4.2	1.16	L1	**1**
192	1	YFE	0.1	10	4.2	1.96	L1	**1**
	2	YFE	0.1	10	4.2	1.96	L1	**1**
	3	YFE	0.1	10	4.2	1.96	L1	**1**
193	1	YFE	0.1	10	4.2	2.36	L1	**1**
	2	YFE	0.1	10	4.2	2.36	L1	**1**
	3	YFE	0.1	10	4.2	2.36	L1	**1**
194	1	YFE	0.1	10	4.2	0.76	H2	**1**
	2	YFE	0.1	10	4.2	0.76	H2	**1**
	3	YFE	0.1	10	4.2	0.76	H2	**1**
195	1	YFE	0.1	10	4.2	1.16	H2	**1**
	2	YFE	0.1	10	4.2	1.16	H2	**1**
	3	YFE	0.1	10	4.2	1.16	H2	**1**
196	1	YFE	0.1	10	4.2	1.96	H2	**1**
	2	YFE	0.1	10	4.2	1.96	H2	**1**
	3	YFE	0.1	10	4.2	1.96	H2	**1**
197	1	YFE	0.1	10	4.2	2.36	H2	**1**
	2	YFE	0.1	10	4.2	2.36	H2	**1**
	3	YFE	0.1	10	4.2	2.36	H2	**1**
198	1	Stout A Botella 1	4.2	31	4.27	1.37	H2	**1**
	2	Stout A Botella 1	4.2	31	4.27	1.37	H2	**1**
	3	Stout A Botella 2	4.2	31	4.27	1.37	H2	**1**
	4	Stout A Botella 2	4.2	31	4.27	1.37	H2	**1**
199	1	Stout A Botella 1	4.2	31	4.27	1.37	B1	**0**
	2	Stout A Botella 1	4.2	31	4.27	1.37	B1	**1**
	3	Stout A Botella 2	4.2	31	4.27	1.37	B1	**0**
	4	Stout A Botella 2	4.2	31	4.27	1.37	B1	**0**
200	1	Stout A Botella 1	4.2	31	4.27	1.37	B2	**0**
	2	Stout A Botella 1	4.2	31	4.27	1.37	B2	**1**
	3	Stout A Botella 2	4.2	31	4.27	1.37	B2	**0**
	4	Stout A Botella 2	4.2	31	4.27	1.37	B2	**0**
201	1	Stout A Botella 1	4.2	31	4.27	1.37	B6	**1**
	2	Stout A Botella 1	4.2	31	4.27	1.37	B6	**1**
	3	Stout A Botella 2	4.2	31	4.27	1.37	B6	**1**

	4	Stout A Botella 2	4.2	31	4.27	1.37	B6	**1**
202	1	Stout A Botella 1	4.2	31	4.27	1.37	F2	**0**
	2	Stout A Botella 1	4.2	31	4.27	1.37	F2	**0**
	3	Stout A Botella 2	4.2	31	4.27	1.37	F2	**1**
	4	Stout A Botella 2	4.2	31	4.27	1.37	F2	**0**
203	1	Stout A Botella 1	4.2	31	4.27	1.37	216	**1**
	2	Stout A Botella 1	4.2	31	4.27	1.37	216	**1**
	3	Stout A Botella 2	4.2	31	4.27	1.37	216	**1**
	4	Stout A Botella 2	4.2	31	4.27	1.37	216	**1**
204	1	Stout A Botella 1	4.2	31	4.27	1.37	L1	**1**
	2	Stout A Botella 1	4.2	31	4.27	1.37	L1	**1**
	3	Stout A Botella 2	4.2	31	4.27	1.37	L1	**1**
	4	Stout A Botella 2	4.2	31	4.27	1.37	L1	**1**
205	1	Stout A Botella 1	4.2	31	4.27	1.37	D1	**1**
	2	Stout A Botella 1	4.2	31	4.27	1.37	D1	**1**
	3	Stout A Botella 2	4.2	31	4.27	1.37	D1	**1**
	4	Stout A Botella 2	4.2	31	4.27	1.37	D1	**1**
206	1	Stout B Botella 1	9	44	4.37	1.26	H2	**1**
	2	Stout B Botella 1	9	44	4.37	1.26	H2	**1**
	3	Stout B Botella 2	9	44	4.37	1.26	H2	**1**
	4	Stout B Botella 2	9	44	4.37	1.26	H2	**1**
207	1	Stout B Botella 1	9	44	4.37	1.26	B1	**0**
	2	Stout B Botella 1	9	44	4.37	1.26	B1	**0**
	3	Stout B Botella 2	9	44	4.37	1.26	B1	**0**
	4	Stout B Botella 2	9	44	4.37	1.26	B1	**0**
208	1	Stout B Botella 1	9	44	4.37	1.26	B2	**0**
	2	Stout B Botella 1	9	44	4.37	1.26	B2	**0**
	3	Stout B Botella 2	9	44	4.37	1.26	B2	**0**
	4	Stout B Botella 2	9	44	4.37	1.26	B2	**0**
209	1	Stout B Botella 1	9	44	4.37	1.26	B6	**0**
	2	Stout B Botella 1	9	44	4.37	1.26	B6	**0**
	3	Stout B Botella 2	9	44	4.37	1.26	B6	**0**
	4	Stout B Botella 2	9	44	4.37	1.26	B6	**0**
210	1	Stout B Botella 1	9	44	4.37	1.26	F2	**0**
	2	Stout B Botella 1	9	44	4.37	1.26	F2	**0**
	3	Stout B Botella 2	9	44	4.37	1.26	F2	**1**
	4	Stout B Botella 2	9	44	4.37	1.26	F2	**0**
211	1	Stout B Botella 1	9	44	4.37	1.26	216	**1**
	2	Stout B Botella 1	9	44	4.37	1.26	216	**1**
	3	Stout B Botella 2	9	44	4.37	1.26	216	**1**
	4	Stout B Botella 2	9	44	4.37	1.26	216	**1**
212	1	Stout B Botella 1	9	44	4.37	1.26	L1	**1**
	2	Stout B Botella 1	9	44	4.37	1.26	L1	**1**
	3	Stout B Botella 2	9	44	4.37	1.26	L1	**1**
	4	Stout B Botella 2	9	44	4.37	1.26	L1	**1**
213	1	Porter A Botella 1	5.9	22	4.08	1.31	H2	**1**
	2	Porter A Botella 1	5.9	22	4.08	1.31	H2	**1**
	3	Porter A Botella 2	5.9	22	4.08	1.31	H2	**1**
	4	Porter A Botella 2	5.9	22	4.08	1.31	H2	**1**
214	1	Porter A Botella 1	5.9	22	4.08	1.31	B1	**0**
	2	Porter A Botella 1	5.9	22	4.08	1.31	B1	**0**
	3	Porter A Botella 2	5.9	22	4.08	1.31	B1	**0**
	4	Porter A Botella 2	5.9	22	4.08	1.31	B1	**0**
215	1	Porter A Botella 1	5.9	22	4.08	1.31	B2	**1**
	2	Porter A Botella 1	5.9	22	4.08	1.31	B2	**1**
	3	Porter A Botella 2	5.9	22	4.08	1.31	B2	**0**
	4	Porter A Botella 2	5.9	22	4.08	1.31	B2	**1**
216	1	Porter A Botella 1	5.9	22	4.08	1.31	B6	**1**
	2	Porter A Botella 1	5.9	22	4.08	1.31	B6	**0**
	3	Porter A Botella 2	5.9	22	4.08	1.31	B6	**1**
	4	Porter A Botella 2	5.9	22	4.08	1.31	B6	**1**
217	1	Porter A Botella 1	5.9	22	4.08	1.31	F2	**1**
	2	Porter A Botella 1	5.9	22	4.08	1.31	F2	**0**
	3	Porter A Botella 2	5.9	22	4.08	1.31	F2	**1**
	4	Porter A Botella 2	5.9	22	4.08	1.31	F2	**1**
218	1	Porter A Botella 1	5.9	22	4.08	1.31	216	**1**
	2	Porter A Botella 1	5.9	22	4.08	1.31	216	**1**
	3	Porter A Botella 2	5.9	22	4.08	1.31	216	**1**

	4	Porter A Botella 2	5.9	22	4.08	1.31	216	**1**
219	1	Porter A Botella 1	5.9	22	4.08	1.31	L1	**1**
	2	Porter A Botella 1	5.9	22	4.08	1.31	L1	**1**
	3	Porter A Botella 2	5.9	22	4.08	1.31	L1	**1**
	4	Porter A Botella 2	5.9	22	4.08	1.31	L1	**1**
220	1	Porter A Botella 1	5.9	22	4.08	1.31	D1	**1**
	2	Porter A Botella 1	5.9	22	4.08	1.31	D1	**1**
	3	Porter A Botella 2	5.9	22	4.08	1.31	D1	**1**
	4	Porter A Botella 2	5.9	22	4.08	1.31	D1	**1**
221	1	Porter B Botella 1	5	29	4.14	0.18	H2	**1**
	2	Porter B Botella 1	5	29	4.14	0.18	H2	**1**
	3	Porter B Botella 2	5	29	4.14	0.18	H2	**1**
	4	Porter B Botella 2	5	29	4.14	0.18	H2	**1**
222	1	Porter B Botella 1	5	29	4.14	0.18	B1	**0**
	2	Porter B Botella 1	5	29	4.14	0.18	B1	**0**
	3	Porter B Botella 2	5	29	4.14	0.18	B1	**0**
	4	Porter B Botella 2	5	29	4.14	0.18	B1	**0**
223	1	Porter B Botella 1	5	29	4.14	0.18	B2	**1**
	2	Porter B Botella 1	5	29	4.14	0.18	B2	**1**
	3	Porter B Botella 2	5	29	4.14	0.18	B2	**0**
	4	Porter B Botella 2	5	29	4.14	0.18	B2	**1**
224	1	Porter B Botella 1	5	29	4.14	0.18	B6	**1**
	2	Porter B Botella 1	5	29	4.14	0.18	B6	**1**
	3	Porter B Botella 2	5	29	4.14	0.18	B6	**0**
	4	Porter B Botella 2	5	29	4.14	0.18	B6	**1**
225	1	Porter B Botella 1	5	29	4.14	0.18	F2	**0**
	2	Porter B Botella 1	5	29	4.14	0.18	F2	**0**
	3	Porter B Botella 2	5	29	4.14	0.18	F2	**0**
	4	Porter B Botella 2	5	29	4.14	0.18	F2	**0**
226	1	Porter B Botella 1	5	29	4.14	0.18	216	**1**
	2	Porter B Botella 1	5	29	4.14	0.18	216	**1**
	3	Porter B Botella 2	5	29	4.14	0.18	216	**1**
	4	Porter B Botella 2	5	29	4.14	0.18	216	**1**
227	1	Porter B Botella 1	5	29	4.14	0.18	L1	**1**
	2	Porter B Botella 1	5	29	4.14	0.18	L1	**1**
	3	Porter B Botella 2	5	29	4.14	0.18	L1	**1**
	4	Porter B Botella 2	5	29	4.14	0.18	L1	**1**
228	1	Porter B Botella 1	5	29	4.14	0.18	D1	**1**
	2	Porter B Botella 1	5	29	4.14	0.18	D1	**1**
	3	Porter B Botella 2	5	29	4.14	0.18	D1	**1**
	4	Porter B Botella 2	5	29	4.14	0.18	D1	**1**
229	1	Brown Ale A Bot1	4.3	21	4.17	0.74	H2	**1**
	2	Brown Ale A Bot1	4.3	21	4.17	0.74	H2	**1**
	3	Brown Ale A Bot2	4.3	21	4.17	0.74	H2	**1**
	4	Brown Ale A Bot2	4.3	21	4.17	0.74	H2	**1**
230	1	Brown Ale A Bot1	4.3	21	4.17	0.74	B1	**0**
	2	Brown Ale A Bot1	4.3	21	4.17	0.74	B1	**1**
	3	Brown Ale A Bot2	4.3	21	4.17	0.74	B1	**0**
	4	Brown Ale A Bot2	4.3	21	4.17	0.74	B1	**0**
231	1	Brown Ale A Bot1	4.3	21	4.17	0.74	B2	**0**
	2	Brown Ale A Bot1	4.3	21	4.17	0.74	B2	**1**
	3	Brown Ale A Bot2	4.3	21	4.17	0.74	B2	**0**
	4	Brown Ale A Bot2	4.3	21	4.17	0.74	B2	**0**
232	1	Brown Ale A Bot1	4.3	21	4.17	0.74	B6	**0**
	2	Brown Ale A Bot1	4.3	21	4.17	0.74	B6	**0**
	3	Brown Ale A Bot2	4.3	21	4.17	0.74	B6	**0**
	4	Brown Ale A Bot2	4.3	21	4.17	0.74	B6	**0**
233	1	Brown Ale A Bot1	4.3	21	4.17	0.74	F2	**0**
	2	Brown Ale A Bot1	4.3	21	4.17	0.74	F2	**0**
	3	Brown Ale A Bot2	4.3	21	4.17	0.74	F2	**0**
	4	Brown Ale A Bot2	4.3	21	4.17	0.74	F2	**0**
234	1	Brown Ale A Bot1	4.3	21	4.17	0.74	216	**1**
	2	Brown Ale A Bot1	4.3	21	4.17	0.74	216	**1**
	3	Brown Ale A Bot2	4.3	21	4.17	0.74	216	**1**
	4	Brown Ale A Bot2	4.3	21	4.17	0.74	216	**1**
235	1	Brown Ale A Bot1	4.3	21	4.17	0.74	L1	**1**
	2	Brown Ale A Bot1	4.3	21	4.17	0.74	L1	**1**
	3	Brown Ale A Bot2	4.3	21	4.17	0.74	L1	**1**

	4	Brown Ale A Bot2	4.3	21	4.17	0.74	L1	**1**
236	1	Brown Ale A Bot1	4.3	21	4.17	0.74	D1	**1**
	2	Brown Ale A Bot1	4.3	21	4.17	0.74	D1	**1**
	3	Brown Ale A Bot2	4.3	21	4.17	0.74	D1	**1**
	4	Brown Ale A Bot2	4.3	21	4.17	0.74	D1	**1**
237	1	Brown Ale B Bot 1	5.2	44	4.46	1.45	H2	**1**
	2	Brown Ale B Bot 1	5.2	44	4.46	1.45	H2	**1**
	3	Brown Ale B Bot 2	5.2	44	4.46	1.45	H2	**1**
	4	Brown Ale B Bot 2	5.2	44	4.46	1.45	H2	**1**
238	1	Brown Ale B Bot 1	5.2	44	4.46	1.45	B1	**0**
	2	Brown Ale B Bot 1	5.2	44	4.46	1.45	B1	**1**
	3	Brown Ale B Bot 2	5.2	44	4.46	1.45	B1	**0**
	4	Brown Ale B Bot 2	5.2	44	4.46	1.45	B1	**0**
239	1	Brown Ale B Bot 1	5.2	44	4.46	1.45	B2	**1**
	2	Brown Ale B Bot 1	5.2	44	4.46	1.45	B2	**1**
	3	Brown Ale B Bot 2	5.2	44	4.46	1.45	B2	**1**
	4	Brown Ale B Bot 2	5.2	44	4.46	1.45	B2	**1**
240	1	Brown Ale B Bot 1	5.2	44	4.46	1.45	B6	**1**
	2	Brown Ale B Bot 1	5.2	44	4.46	1.45	B6	**1**
	3	Brown Ale B Bot 2	5.2	44	4.46	1.45	B6	**1**
	4	Brown Ale B Bot 2	5.2	44	4.46	1.45	B6	**1**
241	1	Brown Ale B Bot 1	5.2	44	4.46	1.45	F2	**0**
	2	Brown Ale B Bot 1	5.2	44	4.46	1.45	F2	**0**
	3	Brown Ale B Bot 2	5.2	44	4.46	1.45	F2	**1**
	4	Brown Ale B Bot 2	5.2	44	4.46	1.45	F2	**0**
242	1	Brown Ale B Bot 1	5.2	44	4.46	1.45	216	**1**
	2	Brown Ale B Bot 1	5.2	44	4.46	1.45	216	**1**
	3	Brown Ale B Bot 2	5.2	44	4.46	1.45	216	**1**
	4	Brown Ale B Bot 2	5.2	44	4.46	1.45	216	**1**
243	1	Brown Ale B Bot 1	5.2	44	4.46	1.45	L1	**1**
	2	Brown Ale B Bot 1	5.2	44	4.46	1.45	L1	**1**
	3	Brown Ale B Bot 2	5.2	44	4.46	1.45	L1	**1**
	4	Brown Ale B Bot 2	5.2	44	4.46	1.45	L1	**1**
244	1	Brown Ale B Bot 1	5.2	44	4.46	1.45	D1	**1**
	2	Brown Ale B Bot 1	5.2	44	4.46	1.45	D1	**1**
	3	Brown Ale B Bot 2	5.2	44	4.46	1.45	D1	**1**
	4	Brown Ale B Bot 2	5.2	44	4.46	1.45	D1	**1**
245	1	Amber Ale A Bot 1	5.5	24	4.25	0.55	H2	**1**
	2	Amber Ale A Bot 1	5.5	24	4.25	0.55	H2	**1**
	3	Amber Ale A Bot 2	5.5	24	4.25	0.55	H2	**1**
	4	Amber Ale A Bot 2	5.5	24	4.25	0.55	H2	**1**
246	1	Amber Ale A Bot 1	5.5	24	4.25	0.55	B1	**0**
	2	Amber Ale A Bot 1	5.5	24	4.25	0.55	B1	**1**
	3	Amber Ale A Bot 2	5.5	24	4.25	0.55	B1	**0**
	4	Amber Ale A Bot 2	5.5	24	4.25	0.55	B1	**0**
247	1	Amber Ale A Bot 1	5.5	24	4.25	0.55	B2	**1**
	2	Amber Ale A Bot 1	5.5	24	4.25	0.55	B2	**1**
	3	Amber Ale A Bot 2	5.5	24	4.25	0.55	B2	**1**
	4	Amber Ale A Bot 2	5.5	24	4.25	0.55	B2	**1**
248	1	Amber Ale A Bot 1	5.5	24	4.25	0.55	B6	**1**
	2	Amber Ale A Bot 1	5.5	24	4.25	0.55	B6	**1**
	3	Amber Ale A Bot 2	5.5	24	4.25	0.55	B6	**1**
	4	Amber Ale A Bot 2	5.5	24	4.25	0.55	B6	**1**
249	1	Amber Ale A Bot 1	5.5	24	4.25	0.55	F2	**1**
	2	Amber Ale A Bot 1	5.5	24	4.25	0.55	F2	**1**
	3	Amber Ale A Bot 2	5.5	24	4.25	0.55	F2	**1**
	4	Amber Ale A Bot 2	5.5	24	4.25	0.55	F2	**1**
250	1	Amber Ale A Bot 1	5.5	24	4.25	0.55	216	**1**
	2	Amber Ale A Bot 1	5.5	24	4.25	0.55	216	**1**
	3	Amber Ale A Bot 2	5.5	24	4.25	0.55	216	**1**
	4	Amber Ale A Bot 2	5.5	24	4.25	0.55	216	**1**
251	1	Amber Ale A Bot 1	5.5	24	4.25	0.55	L1	**1**
	2	Amber Ale A Bot 1	5.5	24	4.25	0.55	L1	**1**
	3	Amber Ale A Bot 2	5.5	24	4.25	0.55	L1	**1**
	4	Amber Ale A Bot 2	5.5	24	4.25	0.55	L1	**1**
252	1	Amber Ale A Bot 1	5.5	24	4.25	0.55	D1	**1**
	2	Amber Ale A Bot 1	5.5	24	4.25	0.55	D1	**1**
	3	Amber Ale A Bot 2	5.5	24	4.25	0.55	D1	**1**

	4	Amber Ale A Bot 2	5.5	24	4.25	0.55	D1	**1**
253	1	Amber Ale B Bot 1	5.8	31	4.26	0.71	H2	**1**
	2	Amber Ale B Bot 1	5.8	31	4.26	0.71	H2	**1**
	3	Amber Ale B Bot 2	5.8	31	4.26	0.71	H2	**1**
	4	Amber Ale B Bot 2	5.8	31	4.26	0.71	H2	**1**
254	1	Amber Ale B Bot 1	5.8	31	4.26	0.71	B1	**0**
	2	Amber Ale B Bot 1	5.8	31	4.26	0.71	B1	**0**
	3	Amber Ale B Bot 2	5.8	31	4.26	0.71	B1	**1**
	4	Amber Ale B Bot 2	5.8	31	4.26	0.71	B1	**0**
255	1	Amber Ale B Bot 1	5.8	31	4.26	0.71	B2	**1**
	2	Amber Ale B Bot 1	5.8	31	4.26	0.71	B2	**1**
	3	Amber Ale B Bot 2	5.8	31	4.26	0.71	B2	**1**
	4	Amber Ale B Bot 2	5.8	31	4.26	0.71	B2	**1**
256	1	Amber Ale B Bot 1	5.8	31	4.26	0.71	B6	**1**
	2	Amber Ale B Bot 1	5.8	31	4.26	0.71	B6	**1**
	3	Amber Ale B Bot 2	5.8	31	4.26	0.71	B6	**1**
	4	Amber Ale B Bot 2	5.8	31	4.26	0.71	B6	**1**
257	1	Amber Ale B Bot 1	5.8	31	4.26	0.71	F2	**0**
	2	Amber Ale B Bot 1	5.8	31	4.26	0.71	F2	**0**
	3	Amber Ale B Bot 2	5.8	31	4.26	0.71	F2	**1**
	4	Amber Ale B Bot 2	5.8	31	4.26	0.71	F2	**0**
258	1	Amber Ale B Bot 1	5.8	31	4.26	0.71	216	**1**
	2	Amber Ale B Bot 1	5.8	31	4.26	0.71	216	**1**
	3	Amber Ale B Bot 2	5.8	31	4.26	0.71	216	**1**
	4	Amber Ale B Bot 2	5.8	31	4.26	0.71	216	**1**
259	1	Amber Ale B Bot 1	5.8	31	4.26	0.71	L1	**1**
	2	Amber Ale B Bot 1	5.8	31	4.26	0.71	L1	**1**
	3	Amber Ale B Bot 2	5.8	31	4.26	0.71	L1	**1**
	4	Amber Ale B Bot 2	5.8	31	4.26	0.71	L1	**1**
260	1	Amber Ale B Bot 1	5.8	31	4.26	0.71	D1	**1**
	2	Amber Ale B Bot 1	5.8	31	4.26	0.71	D1	**1**
	3	Amber Ale B Bot 2	5.8	31	4.26	0.71	D1	**1**
	4	Amber Ale B Bot 2	5.8	31	4.26	0.71	D1	**1**
261	1	IPA A Botella 1	7.2	74	4.32	1.73	H2	**1**
	2	IPA A Botella 1	7.2	74	4.32	1.73	H2	**1**
	3	IPA A Botella 2	7.2	74	4.32	1.73	H2	**1**
	4	IPA A Botella 2	7.2	74	4.32	1.73	H2	**1**
262	1	IPA A Botella 1	7.2	74	4.32	1.73	B1	**0**
	2	IPA A Botella 1	7.2	74	4.32	1.73	B1	**0**
	3	IPA A Botella 2	7.2	74	4.32	1.73	B1	**0**
	4	IPA A Botella 2	7.2	74	4.32	1.73	B1	**0**
263	1	IPA A Botella 1	7.2	74	4.32	1.73	B2	**0**
	2	IPA A Botella 1	7.2	74	4.32	1.73	B2	**0**
	3	IPA A Botella 2	7.2	74	4.32	1.73	B2	**0**
	4	IPA A Botella 2	7.2	74	4.32	1.73	B2	**0**
264	1	IPA A Botella 1	7.2	74	4.32	1.73	B6	**0**
	2	IPA A Botella 1	7.2	74	4.32	1.73	B6	**0**
	3	IPA A Botella 2	7.2	74	4.32	1.73	B6	**0**
	4	IPA A Botella 2	7.2	74	4.32	1.73	B6	**0**
265	1	IPA A Botella 1	7.2	74	4.32	1.73	F2	**0**
	2	IPA A Botella 1	7.2	74	4.32	1.73	F2	**0**
	3	IPA A Botella 2	7.2	74	4.32	1.73	F2	**0**
	4	IPA A Botella 2	7.2	74	4.32	1.73	F2	**0**
266	1	IPA A Botella 1	7.2	74	4.32	1.73	216	**1**
	2	IPA A Botella 1	7.2	74	4.32	1.73	216	**1**
	3	IPA A Botella 2	7.2	74	4.32	1.73	216	**1**
	4	IPA A Botella 2	7.2	74	4.32	1.73	216	**1**
267	1	IPA A Botella 1	7.2	74	4.32	1.73	L1	**1**
	2	IPA A Botella 1	7.2	74	4.32	1.73	L1	**1**
	3	IPA A Botella 2	7.2	74	4.32	1.73	L1	**1**
	4	IPA A Botella 2	7.2	74	4.32	1.73	L1	**1**
267	1	IPA A Botella 1	7.2	74	4.32	1.73	D1	**0**
	2	IPA A Botella 1	7.2	74	4.32	1.73	D1	**1**
	3	IPA A Botella 2	7.2	74	4.32	1.73	D1	**0**
	4	IPA A Botella 2	7.2	74	4.32	1.73	D1	**0**
269	1	IPA B Botella 1	8.5	75	4.7	0.92	H2	**1**
	2	IPA B Botella 1	8.5	75	4.7	0.92	H2	**1**
	3	IPA B Botella 2	8.5	75	4.7	0.92	H2	**1**

	4	IPA B Botella 2	8.5	75	4.7	0.92	H2	**1**
270	1	IPA B Botella 1	8.5	75	4.7	0.92	B1	**0**
	2	IPA B Botella 1	8.5	75	4.7	0.92	B1	**0**
	3	IPA B Botella 2	8.5	75	4.7	0.92	B1	**0**
	4	IPA B Botella 2	8.5	75	4.7	0.92	B1	**0**
271	1	IPA B Botella 1	8.5	75	4.7	0.92	B2	**0**
	2	IPA B Botella 1	8.5	75	4.7	0.92	B2	**0**
	3	IPA B Botella 2	8.5	75	4.7	0.92	B2	**0**
	4	IPA B Botella 2	8.5	75	4.7	0.92	B2	**0**
272	1	IPA B Botella 1	8.5	75	4.7	0.92	B6	**0**
	2	IPA B Botella 1	8.5	75	4.7	0.92	B6	**0**
	3	IPA B Botella 2	8.5	75	4.7	0.92	B6	**0**
	4	IPA B Botella 2	8.5	75	4.7	0.92	B6	**0**
273	1	IPA B Botella 1	8.5	75	4.7	0.92	F2	**0**
	2	IPA B Botella 1	8.5	75	4.7	0.92	F2	**0**
	3	IPA B Botella 2	8.5	75	4.7	0.92	F2	**0**
	4	IPA B Botella 2	8.5	75	4.7	0.92	F2	**0**
274	1	IPA B Botella 1	8.5	75	4.7	0.92	216	**1**
	2	IPA B Botella 1	8.5	75	4.7	0.92	216	**1**
	3	IPA B Botella 2	8.5	75	4.7	0.92	216	**1**
	4	IPA B Botella 2	8.5	75	4.7	0.92	216	**1**
275	1	IPA B Botella 1	8.5	75	4.7	0.92	L1	**1**
	2	IPA B Botella 1	8.5	75	4.7	0.92	L1	**1**
	3	IPA B Botella 2	8.5	75	4.7	0.92	L1	**1**
	4	IPA B Botella 2	8.5	75	4.7	0.92	L1	**1**
276	1	IPA B Botella 1	8.5	75	4.7	0.92	D1	**0**
	2	IPA B Botella 1	8.5	75	4.7	0.92	D1	**0**
	3	IPA B Botella 2	8.5	75	4.7	0.92	D1	**0**
	4	IPA B Botella 2	8.5	75	4.7	0.92	D1	**1**
277	1	Strong Ale A Bot 1	6.5	16	4.2	2.65	H2	**1**
	2	Strong Ale A Bot 1	6.5	16	4.2	2.65	H2	**1**
	3	Strong Ale A Bot 2	6.5	16	4.2	2.65	H2	**1**
	4	Strong Ale A Bot 2	6.5	16	4.2	2.65	H2	**1**
278	1	Strong Ale A Bot 1	6.5	16	4.2	2.65	B1	**0**
	2	Strong Ale A Bot 1	6.5	16	4.2	2.65	B1	**0**
	3	Strong Ale A Bot 2	6.5	16	4.2	2.65	B1	**1**
	4	Strong Ale A Bot 2	6.5	16	4.2	2.65	B1	**0**
279	1	Strong Ale A Bot 1	6.5	16	4.2	2.65	B2	**1**
	2	Strong Ale A Bot 1	6.5	16	4.2	2.65	B2	**1**
	3	Strong Ale A Bot 2	6.5	16	4.2	2.65	B2	**1**
	4	Strong Ale A Bot 2	6.5	16	4.2	2.65	B2	**1**
280	1	Strong Ale A Bot 1	6.5	16	4.2	2.65	B6	**1**
	2	Strong Ale A Bot 1	6.5	16	4.2	2.65	B6	**1**
	3	Strong Ale A Bot 2	6.5	16	4.2	2.65	B6	**1**
	4	Strong Ale A Bot 2	6.5	16	4.2	2.65	B6	**1**
281	1	Strong Ale A Bot 1	6.5	16	4.2	2.65	F2	**0**
	2	Strong Ale A Bot 1	6.5	16	4.2	2.65	F2	**0**
	3	Strong Ale A Bot 2	6.5	16	4.2	2.65	F2	**1**
	4	Strong Ale A Bot 2	6.5	16	4.2	2.65	F2	**0**
282	1	Strong Ale A Bot 1	6.5	16	4.2	2.65	216	**1**
	2	Strong Ale A Bot 1	6.5	16	4.2	2.65	216	**1**
	3	Strong Ale A Bot 2	6.5	16	4.2	2.65	216	**1**
	4	Strong Ale A Bot 2	6.5	16	4.2	2.65	216	**1**
283	1	Strong Ale A Bot 1	6.5	16	4.2	2.65	L1	**1**
	2	Strong Ale A Bot 1	6.5	16	4.2	2.65	L1	**1**
	3	Strong Ale A Bot 2	6.5	16	4.2	2.65	L1	**1**
	4	Strong Ale A Bot 2	6.5	16	4.2	2.65	L1	**1**
284	1	Strong Ale A Bot 1	6.5	16	4.2	2.65	D1	**1**
	2	Strong Ale A Bot 1	6.5	16	4.2	2.65	D1	**1**
	3	Strong Ale A Bot 2	6.5	16	4.2	2.65	D1	**1**
	4	Strong Ale A Bot 2	6.5	16	4.2	2.65	D1	**1**
285	1	Strong Ale B Bot 1	10	25	4.44	1.73	H2	**1**
	2	Strong Ale B Bot 1	10	25	4.44	1.73	H2	**1**
	3	Strong Ale B Bot 2	10	25	4.44	1.73	H2	**1**
	4	Strong Ale B Bot 2	10	25	4.44	1.73	H2	**1**
286	1	Strong Ale B Bot 1	10	25	4.44	1.73	B1	**0**
	2	Strong Ale B Bot 1	10	25	4.44	1.73	B1	**0**
	3	Strong Ale B Bot 2	10	25	4.44	1.73	B1	**0**

	4	Strong Ale B Bot 2	10	25	4.44	1.73	B1	**0**
287	1	Strong Ale B Bot 1	10	25	4.44	1.73	B2	**0**
	2	Strong Ale B Bot 1	10	25	4.44	1.73	B2	**0**
	3	Strong Ale B Bot 2	10	25	4.44	1.73	B2	**0**
	4	Strong Ale B Bot 2	10	25	4.44	1.73	B2	**0**
288	1	Strong Ale B Bot 1	10	25	4.44	1.73	F2	**0**
	2	Strong Ale B Bot 1	10	25	4.44	1.73	F2	**0**
	3	Strong Ale B Bot 2	10	25	4.44	1.73	F2	**1**
	4	Strong Ale B Bot 2	10	25	4.44	1.73	F2	**0**
289	1	Strong Ale B Bot 1	10	25	4.44	1.73	216	**1**
	2	Strong Ale B Bot 1	10	25	4.44	1.73	216	**1**
	3	Strong Ale B Bot 2	10	25	4.44	1.73	216	**1**
	4	Strong Ale B Bot 2	10	25	4.44	1.73	216	**1**
290	1	Strong Ale B Bot 1	10	25	4.44	1.73	L1	**1**
	2	Strong Ale B Bot 1	10	25	4.44	1.73	L1	**1**
	3	Strong Ale B Bot 2	10	25	4.44	1.73	L1	**1**
	4	Strong Ale B Bot 2	10	25	4.44	1.73	L1	**1**
291	1	Strong Ale B Bot 1	10	25	4.44	1.73	D1	**1**
	2	Strong Ale B Bot 1	10	25	4.44	1.73	D1	**1**
	3	Strong Ale B Bot 2	10	25	4.44	1.73	D1	**1**
	4	Strong Ale B Bot 2	10	25	4.44	1.73	D1	**1**
292	1	Non alcoholic A	0.25	11	4.2	2.37	H2	**1**
	2	Non alcoholic A	0.25	11	4.2	2.37	H2	**1**
	3	Non alcoholic A	0.25	11	4.2	2.37	H2	**1**
	4	Non alcoholic A	0.25	11	4.2	2.37	H2	**1**
293	1	Non alcoholic A	0.25	11	4.2	2.37	B1	**1**
	2	Non alcoholic A	0.25	11	4.2	2.37	B1	**1**
	3	Non alcoholic A	0.25	11	4.2	2.37	B1	**1**
	4	Non alcoholic A	0.25	11	4.2	2.37	B1	**1**
294	1	Non alcoholic A	0.25	11	4.2	2.37	B2	**1**
	2	Non alcoholic A	0.25	11	4.2	2.37	B2	**1**
	3	Non alcoholic A	0.25	11	4.2	2.37	B2	**1**
	4	Non alcoholic A	0.25	11	4.2	2.37	B2	**1**
295	1	Non alcoholic A	0.25	11	4.2	2.37	B6	**1**
	2	Non alcoholic A	0.25	11	4.2	2.37	B6	**1**
	3	Non alcoholic A	0.25	11	4.2	2.37	B6	**1**
	4	Non alcoholic A	0.25	11	4.2	2.37	B6	**1**
296	1	Non alcoholic A	0.25	11	4.2	2.37	F2	**1**
	2	Non alcoholic A	0.25	11	4.2	2.37	F2	**1**
	3	Non alcoholic A	0.25	11	4.2	2.37	F2	**1**
	4	Non alcoholic A	0.25	11	4.2	2.37	F2	**1**
297	1	Non alcoholic A	0.25	11	4.2	2.37	216	**1**
	2	Non alcoholic A	0.25	11	4.2	2.37	216	**1**
	3	Non alcoholic A	0.25	11	4.2	2.37	216	**1**
	4	Non alcoholic A	0.25	11	4.2	2.37	216	**1**
298	1	Non alcoholic A	0.25	11	4.2	2.37	L1	**1**
	2	Non alcoholic A	0.25	11	4.2	2.37	L1	**1**
	3	Non alcoholic A	0.25	11	4.2	2.37	L1	**1**
	4	Non alcoholic A	0.25	11	4.2	2.37	L1	**1**
299	1	Non alcoholic A	0.25	11	4.2	2.37	D1	**1**
	2	Non alcoholic A	0.25	11	4.2	2.37	D1	**1**
	3	Non alcoholic A	0.25	11	4.2	2.37	D1	**1**
	4	Non alcoholic A	0.25	11	4.2	2.37	D1	**1**
300	1	Wheat A Botella 1	5.5	15	4.29	1	H2	**1**
	2	Wheat A Botella 1	5.5	15	4.29	1	H2	**1**
	3	Wheat A Botella 2	5.5	15	4.29	1	H2	**1**
	4	Wheat A Botella 2	5.5	15	4.29	1	H2	**1**
301	1	Wheat A Botella 1	5.5	15	4.29	1	B1	**0**
	2	Wheat A Botella 1	5.5	15	4.29	1	B1	**1**
	3	Wheat A Botella 2	5.5	15	4.29	1	B1	**0**
	4	Wheat A Botella 2	5.5	15	4.29	1	B1	**0**
302	1	Wheat A Botella 1	5.5	15	4.29	1	B2	**1**
	2	Wheat A Botella 1	5.5	15	4.29	1	B2	**1**
	3	Wheat A Botella 2	5.5	15	4.29	1	B2	**1**
	4	Wheat A Botella 2	5.5	15	4.29	1	B2	**1**
303	1	Wheat A Botella 1	5.5	15	4.29	1	B6	**0**
	2	Wheat A Botella 1	5.5	15	4.29	1	B6	**0**
	3	Wheat A Botella 2	5.5	15	4.29	1	B6	**0**

	4	Wheat A Botella 2	5.5	15	4.29	1	B6	**1**
304	1	Wheat A Botella 1	5.5	15	4.29	1	F2	**0**
	2	Wheat A Botella 1	5.5	15	4.29	1	F2	**1**
	3	Wheat A Botella 2	5.5	15	4.29	1	F2	**0**
	4	Wheat A Botella 2	5.5	15	4.29	1	F2	**0**
305	1	Wheat A Botella 1	5.5	15	4.29	1	216	**1**
	2	Wheat A Botella 1	5.5	15	4.29	1	216	**1**
	3	Wheat A Botella 2	5.5	15	4.29	1	216	**1**
	4	Wheat A Botella 2	5.5	15	4.29	1	216	**1**
306	1	Wheat A Botella 1	5.5	15	4.29	1	L1	**1**
	2	Wheat A Botella 1	5.5	15	4.29	1	L1	**1**
	3	Wheat A Botella 2	5.5	15	4.29	1	L1	**1**
	4	Wheat A Botella 2	5.5	15	4.29	1	L1	**1**
307	1	Wheat A Botella 1	5.5	15	4.29	1	D1	**1**
	2	Wheat A Botella 1	5.5	15	4.29	1	D1	**1**
	3	Wheat A Botella 2	5.5	15	4.29	1	D1	**1**
	4	Wheat A Botella 2	5.5	15	4.29	1	D1	**1**
308	1	Wheat B Bot 1	3.8	12	3.14	2.37	H2	**1**
	2	Wheat B Bot 1	3.8	12	3.14	2.37	H2	**1**
	3	Wheat B Bot 2	3.8	12	3.14	2.37	H2	**0**
	4	Wheat B Bot 2	3.8	12	3.14	2.37	H2	**1**
309	1	Wheat B Bot 1	3.8	12	3.14	2.37	B1	**0**
	2	Wheat B Bot 1	3.8	12	3.14	2.37	B1	**0**
	3	Wheat B Bot 2	3.8	12	3.14	2.37	B1	**0**
	4	Wheat B Bot 2	3.8	12	3.14	2.37	B1	**0**
310	1	Wheat B Bot 1	3.8	12	3.14	2.37	B2	**0**
	2	Wheat B Bot 1	3.8	12	3.14	2.37	B2	**0**
	3	Wheat B Bot 2	3.8	12	3.14	2.37	B2	**0**
	4	Wheat B Bot 2	3.8	12	3.14	2.37	B2	**0**
311	1	Wheat B Bot 1	3.8	12	3.14	2.37	B6	**1**
	2	Wheat B Bot 1	3.8	12	3.14	2.37	B6	**1**
	3	Wheat B Bot 2	3.8	12	3.14	2.37	B6	**0**
	4	Wheat B Bot 2	3.8	12	3.14	2.37	B6	**1**
312	1	Wheat B Bot 1	3.8	12	3.14	2.37	F2	**0**
	2	Wheat B Bot 1	3.8	12	3.14	2.37	F2	**0**
	3	Wheat B Bot 2	3.8	12	3.14	2.37	F2	**0**
	4	Wheat B Bot 2	3.8	12	3.14	2.37	F2	**0**
313	1	Wheat B Bot 1	3.8	12	3.14	2.37	216	**0**
	2	Wheat B Bot 1	3.8	12	3.14	2.37	216	**0**
	3	Wheat B Bot 2	3.8	12	3.14	2.37	216	**0**
	4	Wheat B Bot 2	3.8	12	3.14	2.37	216	**0**
314	1	Wheat B Bot 1	3.8	12	3.14	2.37	L1	**0**
	2	Wheat B Bot 1	3.8	12	3.14	2.37	L1	**0**
	3	Wheat B Bot 2	3.8	12	3.14	2.37	L1	**0**
	4	Wheat B Bot 2	3.8	12	3.14	2.37	L1	**0**
315	1	Wheat B Bot 1	3.8	12	3.14	2.37	D1	**0**
	2	Wheat B Bot 1	3.8	12	3.14	2.37	D1	**0**
	3	Wheat B Bot 2	3.8	12	3.14	2.37	D1	**0**
	4	Wheat B Bot 2	3.8	12	3.14	2.37	D1	**0**
316	1	Bock A Botella 1	12	25	4.66	1.68	H2	**0**
	2	Bock A Botella 1	12	25	4.66	1.68	H2	**1**
	3	Bock A Botella 2	12	25	4.66	1.68	H2	**0**
	4	Bock A Botella 2	12	25	4.66	1.68	H2	**0**
317	1	Bock A Botella 1	12	25	4.66	1.68	B1	**0**
	2	Bock A Botella 1	12	25	4.66	1.68	B1	**0**
	3	Bock A Botella 2	12	25	4.66	1.68	B1	**0**
	4	Bock A Botella 2	12	25	4.66	1.68	B1	**0**
318	1	Bock A Botella 1	12	25	4.66	1.68	B2	**0**
	2	Bock A Botella 1	12	25	4.66	1.68	B2	**0**
	3	Bock A Botella 2	12	25	4.66	1.68	B2	**0**
	4	Bock A Botella 2	12	25	4.66	1.68	B2	**0**
319	1	Bock A Botella 1	12	25	4.66	1.68	B6	**0**
	2	Bock A Botella 1	12	25	4.66	1.68	B6	**0**
	3	Bock A Botella 2	12	25	4.66	1.68	B6	**0**
	4	Bock A Botella 2	12	25	4.66	1.68	B6	**0**
320	1	Bock A Botella 1	12	25	4.66	1.68	F2	**0**
	2	Bock A Botella 1	12	25	4.66	1.68	F2	**0**
	3	Bock A Botella 2	12	25	4.66	1.68	F2	**0**

	4	Bock A Botella 2	12	25	4.66	1.68	F2	**0**
321	1	Bock A Botella 1	12	25	4.66	1.68	216	**0**
	2	Bock A Botella 1	12	25	4.66	1.68	216	**0**
	3	Bock A Botella 2	12	25	4.66	1.68	216	**0**
	4	Bock A Botella 2	12	25	4.66	1.68	216	**0**
322	1	Bock A Botella 1	12	25	4.66	1.68	L1	**0**
	2	Bock A Botella 1	12	25	4.66	1.68	L1	**1**
	3	Bock A Botella 2	12	25	4.66	1.68	L1	**0**
	4	Bock A Botella 2	12	25	4.66	1.68	L1	**0**
323	1	Bock A Botella 1	12	25	4.66	1.68	D1	**0**
	2	Bock A Botella 1	12	25	4.66	1.68	D1	**0**
	3	Bock A Botella 2	12	25	4.66	1.68	D1	**0**
	4	Bock A Botella 2	12	25	4.66	1.68	D1	**0**
324	1	Bock B Botella 1	7	10	4.23	0.76	H2	**1**
	2	Bock B Botella 1	7	10	4.23	0.76	H2	**1**
	3	Bock B Botella 2	7	10	4.23	0.76	H2	**1**
	4	Bock B Botella 2	7	10	4.23	0.76	H2	**1**
325	1	Bock B Botella 1	7	10	4.23	0.76	B1	**1**
	2	Bock B Botella 1	7	10	4.23	0.76	B1	**1**
	3	Bock B Botella 2	7	10	4.23	0.76	B1	**1**
	4	Bock B Botella 2	7	10	4.23	0.76	B1	**1**
326	1	Bock B Botella 1	7	10	4.23	0.76	B2	**1**
	2	Bock B Botella 1	7	10	4.23	0.76	B2	**1**
	3	Bock B Botella 2	7	10	4.23	0.76	B2	**1**
	4	Bock B Botella 2	7	10	4.23	0.76	B2	**1**
327	1	Bock B Botella 1	7	10	4.23	0.76	B6	**1**
	2	Bock B Botella 1	7	10	4.23	0.76	B6	**1**
	3	Bock B Botella 2	7	10	4.23	0.76	B6	**1**
	4	Bock B Botella 2	7	10	4.23	0.76	B6	**1**
328	1	Bock B Botella 1	7	10	4.23	0.76	F2	**1**
	2	Bock B Botella 1	7	10	4.23	0.76	F2	**1**
	3	Bock B Botella 2	7	10	4.23	0.76	F2	**1**
	4	Bock B Botella 2	7	10	4.23	0.76	F2	**1**
329	1	Bock B Botella 1	7	10	4.23	0.76	216	**1**
	2	Bock B Botella 1	7	10	4.23	0.76	216	**1**
	3	Bock B Botella 2	7	10	4.23	0.76	216	**1**
	4	Bock B Botella 2	7	10	4.23	0.76	216	**1**
330	1	Bock B Botella 1	7	10	4.23	0.76	L1	**1**
	2	Bock B Botella 1	7	10	4.23	0.76	L1	**1**
	3	Bock B Botella 2	7	10	4.23	0.76	L1	**1**
	4	Bock B Botella 2	7	10	4.23	0.76	L1	**1**
331	1	Bock B Botella 1	7	10	4.23	0.76	D1	**1**
	2	Bock B Botella 1	7	10	4.23	0.76	D1	**1**
	3	Bock B Botella 2	7	10	4.23	0.76	D1	**1**
	4	Bock B Botella 2	7	10	4.23	0.76	D1	**1**

Supplementary Table S2: Data for External Model Validation and the observed and predicted probability												
Nº assay	Sample	Beer Style	% ABV	IBU	pH	%YFE	Strain	G/NG results	Observed probability		Predicted probability	
1	Beer 1	Blond Ale	4.5	33	4.68	0.67	L1	1	66.66%	Easy to spoil*	90.4%	Easy to spoil
2			4.5	33	4.68	0.67	D1	1				
3			4.5	33	4.68	0.67	216	1				
4			4.5	33	4.68	0.67	B1	0				
5			4.5	33	4.68	0.67	B2	1				
6			4.5	33	4.68	0.67	B6	0				
7			4.5	33	4.68	0.67	F2	1				
8			4.5	33	4.68	0.67	F1	0				
9			4.5	33	4.68	0.67	H2	1				
10	Beer 2	Non-alcoholic	0.04	30	4.15	2.76	L1	1	100%	Easy to spoil	90.35%	Easy to spoil
11			0.04	30	4.15	2.76	D1	1				
12			0.04	30	4.15	2.76	216	1				
13			0.04	30	4.15	2.76	B1	1				
14			0.04	30	4.15	2.76	B2	1				
15			0.04	30	4.15	2.76	B6	1				
16			0.04	30	4.15	2.76	F2	1				
17			0.04	30	4.15	2.76	F1	1				
18			0.04	30	4.15	2.76	H2	1				
19	Beer 3	Brown Ale	4.7	18	4.5	0.91	L1	1	100%	Easy to spoil	90.33%	Easy to spoil
20			4.7	18	4.5	0.91	D1	1				
21			4.7	18	4.5	0.91	216	1				
22			4.7	18	4.5	0.91	B1	1				
23			4.7	18	4.5	0.91	B2	1				
24			4.7	18	4.5	0.91	B6	1				
25			4.7	18	4.5	0.91	F2	1				
26			4.7	18	4.5	0.91	F1	1				
27			4.7	18	4.5	0.91	H2	1				
28	Beer 4	Lager Helles	4.5	30	4.63	0.24	L1	1	55.55%	Easy to spoil	90.12%	Easy to spoil
29			4.5	30	4.63	0.24	D1	1				
30			4.5	30	4.63	0.24	216	1				
31			4.5	32	4.63	0.24	B1	0				
32			4.5	32	4.63	0.24	B2	0				
33			4.5	32	4.63	0.24	B6	0				
34			4.5	32	4.63	0.24	F2	1				
35			4.5	32	4.63	0.24	F1	0				
36			4.5	32	4.63	0.24	H2	1				
37	Beer 5	IPA	6	30	4.61	0.21	L1	1	55.55%	Easy to spoil	83.59%	Easy to spoil
38			6	30	4.61	0.21	D1	1				
39			6	30	4.61	0.21	216	1				
40			6	30	4.61	0.21	B1	0				
41			6	30	4.61	0.21	B2	0				
42			6	30	4.61	0.21	B6	0				
43			6	30	4.61	0.21	F2	1				
44			6	30	4.61	0.21	F1	0				
45			6	30	4.61	0.21	H2	1				
46	Beer 6	Porter	4.9	33	4.49	0.72	L1	1	88.88%	Easy to spoil	81.81%	Easy to spoil
47			4.9	33	4.49	0.72	D1	1				
48			4.9	33	4.49	0.72	216	1				
49			4.9	33	4.49	0.72	B1	0				
50			4.9	33	4.49	0.72	B2	1				
51			4.9	33	4.49	0.72	B6	1				
52			4.9	33	4.49	0.72	F2	1				
53			4.9	33	4.49	0.72	F1	1				
54			4.9	33	4.49	0.72	H2	1				

55			5.2	30	4.32	0.88	L1	**1**				
56			5.2	30	4.32	0.88	D1	**1**				
57			5.2	30	4.32	0.88	216	**1**				
58			5.2	30	4.32	0.88	B1	**1**				
59	**Beer 7**	**Pale Ale**	5.2	30	4.32	0.88	B2	**1**	100%	**Easy to spoil**	72.88%	**Easy to spoil**
60			5.2	30	4.32	0.88	B6	**1**				
61			5.2	30	4.32	0.88	F2	**1**				
62			5.2	30	4.32	0.88	F1	**1**				
63			5.2	30	4.32	0.88	H2	**1**				
64			7	26	4.46	1.65	L1	**1**				
65			7	26	4.46	1.65	D1	**1**				
66			7	26	4.46	1.65	216	**1**				
67			7	26	4.46	1.65	B1	**0**				
68	**Beer 8**	**Stout**	7	26	4.46	1.65	B2	**1**	77.77%	**Easy to spoil**	72.63%	**Easy to spoil**
69			7	26	4.46	1.65	B6	**0**				
70			7	26	4.46	1.65	F2	**1**				
71			7	26	4.46	1.65	F1	**1**				
72			7	26	4.46	1.65	H2	**1**				
73			5.1	44	4.44	1.63	L1	**1**				
74			5.1	44	4.44	1.63	D1	**1**				
75			5.1	44	4.44	1.63	216	**1**				
76			5.1	44	4.44	1.63	B1	**0**				
77	**Beer 9**	**English IPA**	5.1	44	4.44	1.63	B2	**0**	55.55%	**Easy to spoil**	69.3%	**Easy to spoil**
78			5.1	44	4.44	1.63	B6	**0**				
79			5.1	44	4.44	1.63	F2	**1**				
80			5.1	44	4.44	1.63	F1	**0**				
81			5.1	44	4.44	1.63	H2	**1**				
82			6.5	10	3.47	0.96	L1	**0**				
83			6.5	10	3.47	0.96	D1	**0**				
84			6.5	10	3.47	0.96	216	**0**				
85			6.5	10	3.47	0.96	B1	**0**				
86	**Beer 10**	**Barley Wine**	6.5	10	3.47	0.96	B2	**0**	0%	**Not easy to spoil^**	21.28%	**Not easy to spoil**
87			6.5	10	3.47	0.96	B6	**0**				
88			6.5	10	3.47	0.96	F2	**0**				
89			6.5	10	3.47	0.96	F1	**0**				
90			6.5	10	3.47	0.96	H2	**0**				
*Easy to spoil: The predicted probability is > cut value; ^Not easy to spoil: The predicted probability is < cut value									**% correctly predicted**		100%	
